# CSE-8, a filamentous fungus-specific Shr3-like chaperone, facilitates endoplasmic reticulum exit of chitin synthase CHS-3 (class I) in *Neurospora crassa*

**DOI:** 10.3389/ffunb.2024.1505388

**Published:** 2025-01-24

**Authors:** Samantha Verónica González-Téllez, Meritxell Riquelme

**Affiliations:** Department of Microbiology, Centro de Investigación Científica y de Educación Superior de Ensenada (CICESE), Ensenada, Mexico

**Keywords:** chitin synthases, endoplasmic reticulum, cargo receptor protein, Spitzenkörper, endoplasmic reticulum chaperones, perithecia

## Abstract

Chitin is a crucial structural polysaccharide in fungal cell walls, essential for maintaining cellular plasticity and integrity. Its synthesis is orchestrated by chitin synthases (CHS), a major family of transmembrane proteins. In *Saccharomyces cerevisiae*, the cargo receptor Chs7, belonging to the Shr3-like chaperone family, plays a pivotal role in the exit of Chs3 from the endoplasmic reticulum (ER) and its subsequent activity in the plasma membrane (PM). However, the auxiliary machinery responsible for CHS trafficking in filamentous fungi remains poorly understood. The *Neurospora crassa* genome encodes two orthologues of Chs7: chitin synthase export (CSE) proteins CSE-7 (NCU05720) and CSE-8 (NCU01814), both of which are highly conserved among filamentous fungi. In contrast, yeast forms only possess a single copy CHS export receptor. Previous research highlighted the crucial role of CSE-7 in the localization of CHS-4 at sites of cell wall synthesis, including the Spitzenkörper (SPK) and septa. In this study, CSE-8 was identified as an export protein for CHS-3 (class I). In the *Δcse-8* knockout strain of *N. crassa*, CHS-3-GFP fluorescence was absent from the SPK or septa, indicating that CSE-8 is required for the exit of CHS-3 from the ER. Additionally, sexual development was disrupted in the *Δcse-8* strain, with 20% of perithecia from homozygous crosses exhibiting two ostioles. A *Δcse-7;Δcse-8* double mutant strain showed reduced N-acetylglucosamine (GlcNAc) content and decreased radial growth. Furthermore, the loss of cell polarity and the changes in subcellular distribution of CSE-8-GFP and CHS-3-GFP observed in hyphae under ER stress induced by the addition of tunicamycin and dithiothreitol reinforce the hypothesis that CSE-8 functions as an ER protein. The current evidence suggests that the biogenesis of CHS exclusive to filamentous fungi may involve pathways independent of CSE-mediated receptors.

## 1 Introduction

The fungal cell wall is a dynamic structure essential for maintaining cell integrity. It is primarily composed of structural polysaccharides, including chitin, glucans, and proteins. Chitin, a homopolymer of N-acetylglucosamine (GlcNAc) subunits linked by β-(1,4)-glycosidic bonds, forms a network of linear molecules that confer rigidity and strength to the cell wall (Bowman and Free, 2006; Gow et al., 2017; Kar et al., 2019). The synthesis of chitin is catalyzed by chitin synthases (CHS), which are delivered to synthesis sites via specialized microvesicles known as chitosomes (Bartnicki-Garcia et al., 1978). The CHS family is divided into seven classes based on their amino acid composition and is further categorized into three divisions according to their conserved protein domains (Pacheco-Arjona and Ramirez-Prado, 2014). Division 1 includes classes I, II, and III of CHSs, characterized by a hydrophobic C-terminal domain and a hydrophilic catalytic subdomain in the N-terminal region. Division 2 encompasses classes IV, V, and VII, all sharing a conserved cytochrome b5 catalytic domain. Division 3 comprises class VI CHS, distinguished by a Pfam03142 domain and a signal peptide motif (Riquelme and Bartnicki-García, 2008; Fajardo-Somera et al., 2015). Notably, CHS classes III, V, VI, and VII are unique to filamentous fungi.

In the *Neurospora crassa* genome, there are seven *chs* genes, each encoding a different class of CHS, which play distinct roles during cell development and septum biogenesis (Borkovich et al., 2004; Cabib, 2004; Riquelme and Bartnicki-García, 2008; Cabib and Schmidt, 2013; Fajardo-Somera et al., 2015). All *N. crassa* CHS are transported to the plasma membrane (PM) within chitosomes that accumulate in the core of the Spitzenkörper (SPK) before being secreted (Bartnicki-Garcia, 1987, 2006; Riquelme et al., 2007; Riquelme, 2013; Fajardo-Somera et al., 2015). However, the factors regulating CHS transport and PM activity remain poorly understood, and the machinery involved in the exit of CHS from the endoplasmic reticulum (ER), where they are packaged for transport to the apical zones of the hyphae, is largely unknown. In the model yeast *Saccharomyces cerevisiae*, there are only three CHS classes, with Chs3 (class IV) responsible for synthesizing 90-95% of the chitin (Pammer et al., 1992; Sudoh et al., 1999; Bulik et al., 2003; Orlean, 2012). For Chs3 to exit the ER, it must be palmitoylated by Pfa4 at two catalytic sites, allowing it to associate with Chs7 (Trilla et al., 1999; Lam et al., 2006; González Montoro et al., 2011; Orlean, 2012). A Δ*chs7* strain exhibits a chitin content defect nearly identical to Δ*chs3*, and the deletion of *CHS7* causes Chs3-GFP to accumulate in the ER (Trilla et al., 1999; Orlean, 2012). Thus, Chs7 functions as a chaperone for Chs3. Furthermore, while initially characterized as an ER-resident protein responsible for Chs3 egress to the Golgi apparatus (Trilla et al., 1999), later studies demonstrated that Chs7 does not remain in the ER after Chs3 exit. Instead, it forms a complex with Chs3 that facilitates its proper folding and activity in the PM (Dharwada et al., 2018). In *Candida albicans*, a *CHS7* deletion mutant resulted in reduced chitin levels, morphogenetic alterations, and also attenuated virulence (Sanz et al., 2005).

*S. cerevisiae* Chs7 belongs to a small group of four ER chaperone-like transmembrane proteins known as “Shr3-like” proteins, which include Shr3, Pho86, Gsf2, and Chs7 (Kota and Ljungdahl, 2005). These proteins function as specialized chaperones that prevent the aggregation of PM proteins at the ER by ensuring proper folding (Kota and Ljungdahl, 2005). Unlike other chaperones, “Shr3-like” proteins do not interact with a broad range of cargoes and lack conserved domains common to other chaperone families. Shr3, an ER-resident protein, assists amino acid permeases (AAPs) by utilizing its hydrophilic C-terminal domain to associate with COPII coatomer subunits, facilitating AAP transport through the secretory pathway (Kota and Ljungdahl, 2005). Similarly, Pho86 is an ER protein responsible for packaging the phosphate transporter Pho84 into COPII vesicles for secretory transport (Lau et al., 2000; Shen et al., 2012). Mutations in Gsf2 lead to the accumulation of the hexose transporter Hxt1 at ER exit sites (Sherwood and Carlson, 1999).

The function of Chs7 orthologous proteins in filamentous fungi remained unclear until two orthologues of *S. cerevisiae* Chs7 were identified in *N. crassa*. The first orthologue, CSE-7 (*Chitin Synthase Export chaperone 7*), is located in the ER and tubular vacuoles, where it plays a role in the secretion and biogenesis of CHS-4, a class IV CHS ortholog of the yeast Chs3 (Rico-Ramírez et al., 2018). The *Δcse-7* strain of *N. crassa* did not display significant phenotypic alterations, consistent with the phenotype of the *Δchs-4* strain. In contrast, the *Trichoderma atroviridae Δcse-7* strain exhibited noticeable changes in colony morphology, characterized by stratified mycelium with abundant branching (Kappel et al., 2020). Much of what is known about CHS biogenesis and transport to the apical region and PM comes from studies in yeast (Munro et al., 2001; Preechasuth et al., 2015). Research in *S. cerevisiae* has identified several auxiliary proteins that function as chaperones in the vesicular trafficking of chitosomes (Munro, 2013; Sanz, 2004). This study investigated the role of the second *S. cerevisiae* Chs7 orthologue, CSE-8 (*Chitin Synthase Export chaperone 8*), in CHS trafficking. Our findings provide evidence that CSE-8 is involved in the trafficking of CHS-3 (Class I)-carrying chitosomes, with their transport to septa and SPK being disrupted in the absence of the *cse-8* gene.

## 2 Materials and methods

### 2.1 Molecular constructs

Endogenous labeling of *cse-8* with *gfp* was carried out using the Split Marker method (Smith et al., 2011). The oligonucleotides cse-8/Gly-FW and cse-8/Gly-RV (**Table 1**) were used to directly amplify the ORF of the *cse-8* gene using genomic DNA from *N. crassa* FGSC #988 strain as a template. Likewise, the oligonucleotides FW-lox/3’cse-8 and RV-lox/3’cse-8 (**Table 1**) were used to amplify the 3’ UTR region of the *cse-8* gene. The plasmid pRS416 (Honda and Selker, 2009), was used as a template to amplify the green fluorescent protein gene (*gfp*) fused to 10 glycines and the *hph* gene (hygromycin B conferring resistant gene) using the oligonucleotides loxP-R and 10xGly-F (**Table 1**). Subsequently, fusion PCRs were performed to fuse the PCR products and obtain the constructs *cse-8::gfp::hph* and *hph::cse-8*. The resulting constructs were purified after running them by gel electrophoresis (1% agarose), and conidia of *N. crassa* (FGSC #9718) were transformed with 500 ng of each construct by electroporation in a Biorad Gene Pulser Electroporation using 0.2 mm electroporation cuvettes (600 OHMS, 25 μFD, 1.5 KV).

**Table 1:** Primers used in this study.

Name	Sequence
P1-cse8 FW	CCTCCATATTTACAGGACTTCTGTCG
P2-cse8 RV	GGCCAATCGTCTTCGGTAATGCTG
cse-8/Gly FW	ATGGGCTCAACACAATTTGGCAACTTTCATG
cse-8/Gly RV	CCTCCGCCTCCGCCTCCGCCGCCTCCGCCTGGGAACTGGTTAGGCGGAACC
lox/3'cse-8 FW	TGCTATACGAAGTTATGGATCCGAGCTCGAAGGGCCAGTACAGGTTGAAGTCTCG
lox/3'cse-8 RV	GGGCACGACAAATCGGATTTATGGG
cse-8_3'UTR	CGTCCGCATGTTCTTCTTCCA
chs-1_ORF	CGTCCGCATGTTCTTCTTCCACGT
ORF chs-3 FW	CCGCCTTTGGCTTCATTTCCGTCT
ORF chs-3 RV	CGTGTAGCTTCTCACCGGCAAAGT
FWPccg1	TTCGTTCAAAGCCACATCACTGGG
GFP-F/P5	ATGGTGAGCAAGGGCGAG
GFP-R/P6	CTTGTACAGCTCGTCCATGC
loxP-R	CGAGCTCGGATCCATAACTTCGTATA
10xGly-F	GGCGGAGGCGGCGGAGGCGGAGGC
hph SM-r	TCGCCTCGCTCCAGTCAATGACC
hph SM-f	AAAAAGCCTGAACTCACCGCGACG
chs-5 P1 seq F	CTGACAACACTGCTTCTTAAGTTC

### 2.2 Strains and culture conditions

All fungal or bacterial strains used or generated in this study are listed in **Table 2**. *N. crassa* strains were grown in Vogel’s minimum medium (VMM) supplemented with 1.5% sucrose and 1% agar (Vogel, 1956). For phenotypic characterization, 1x10^5^ conidia were inoculated onto VMM plates supplemented with NaCl (0.8 mM), KCl (0.8 mM), and Congo Red (100 mg/mL) to induce osmotic and cell wall stress. For optimal growth, inoculated flasks and plates were incubated at 30°C (Davis, 2000). For the selection of hygromycin-resistant transformants, conidia were incubated in a recovery solution (1X Vogel’s salts and 2% yeast extract) after electroporation. After 3 hours of incubation, transformed conidia of FGSC #9718 were inoculated onto FGS medium supplemented with hygromycin B (300 μg/mL). The conidia of FGSC #9717 strains were inoculated on FGS plates with or without (for negative controls) histidine (0.25 mg/mL). The FGS medium contains 2% Vogel’s salts, 1% agar, and 10% FGS solution (0.5% fructose, 0.5% glucose, and 20% sorbose). All media components were sterilized by filtration, and the media were autoclaved at 15 lbf/in^2^ for 15 minutes. Synthetic crossing medium (50% SCM 2X solution, 2% sucrose, and 1.5% agar) was used to obtain homokaryotic Δ*cse-8* strains expressing CHS-1-GFP, CHS-3-GFP, or CHS-5-GFP, and a double mutant strain Δ*cse-8;*Δ*cse-7*.

**Table 2:** Strains and plasmids used or generated in this study.

Strain	Genotype	Source
*Escherichia coli*		
DH5^TM^	F^-^ Φ80lacZΔM15 Δ(lacZYA-argF) U169 recA1 endA1 hsdR17(rk^-^,mk^+^) phoA supE44thi-1 gyrA96 relA1 λ^-^	Invitrogen ®
*Neurospora crassa*		
FGSC #4200	*mat A;* wild type	FGSC
FGSC #9717	*his-3::Δmus-51*::*bar^+^*	FGSC
FGSC #9718	*Δmus-51::bar^+^*	FGSC
FGSC #13138	Δ*cse-8; mat A; hyg^r^*	FGSC
NSSG1	Δ*cse-7;* Δ*cse-8; mat a; hyg^r^*	This study
FGSC #13680	Δ*cse-7; mat A; hyg^r^*	FGSC
NSSG2	Δ*cse-8::hph^r^; Δcse-7::hph^r^*	This study
SMRP207	*Pchs-5::chs-5::gfp*; *hph*; *mat a*	Fajardo-Somera et al. 2015
FGSC #13408	*Pchs-1::chs-1::gfp*; *hph*; *mat a*	Sánchez-León et al. 2011
NSSG3	*Pcse-8::gfp::hph; Δmus-51::bar^+^*	This study
NSSG4	*Pchs-1::sgfp::hph; Δcse-8, hyg^r^ , Δmus-51::bar^+^*	This study
NSSG5	*Pchs-5::sgfp::hph; Δcse-8, hyg^r^ , Δmus-51::bar^+^*	This study
NMR3-1	*his3^+^::Pccg-1-chs3-sgfp*	Riquelme et al. 2007
NSSG6	*his3^+^::Pccg-1-chs3-sgfp; Δcse-8*	This study
SMRP302	*his-3 ^+^::Pccg-1::mchfp^+^::ypt-1; Δmus51::bar^+^*	Sánchez-León et al. 2014
FGSC #10159	*Pccg-1::dsred-nca-1::his-3^+^:: Δmus51::bar+*	Bowman et al. 2009
FGSC #11624	*Pccg-1::rfp-grp-78::his-3^+^:: Δmus51::bar+*	Bowman et al. 2009
Plasmids		
pRS416	10xGly*::gfp::hph*	Honda and Selker, 2009

### 2.3 Confocal microscopy and image processing

We used an Olympus SZX12 stereoscopic microscope adapted to a C-HP4 8MP 4K, Full HD C-mount camera and equipped with an Olympus DF PLAPO 1XPF objective to image colony morphology and perithecia. An Olympus Fluoview FV1000 inverted Laser scanning confocal microscope (LSCM) was used to image GFP, dsRED, mCherry, and RFP-tagged proteins, employing 488 nm and 543 nm Lasers. FM4-64, diluted in liquid VMM to a final concentration of 5 µM, was used to stain the NSSG3 strain to identify CSE-8 in subcellular compartments. Nuclei were stained with a stock solution of Hoechst 33258 (100 mg/mL) in distilled water, diluted to a final concentration of 26 µg/mL in filtered PBS buffer (pH=5.2) before use. Stock solutions of 1M 1,4-dithiothreitol (DTT, Sigma-Aldrich) in water and 200 µM tunicamycin (TM, Sigma-Aldrich) in dimethyl sulfoxide (DMSO) were prepared as ER stressors and used at final concentrations of 1.25 µM and 4 µg/mL in VMM, respectively. A stock solution of brefeldin A (BFA, Sigma-Aldrich) in DMSO was prepared at 20 mg/mL and diluted in MMV at a final concentration of 200 µg/mL. This concentration was selected based on prior studies demonstrating its efficacy in disrupting CHS-4-GFP (class III) transport to the SPK in *N. crassa* (Fajardo-Somera et al., 2015). All samples were incubated at 30°C for 15 to 20 minutes before imaging, following the inverted agar method (Hickey et al., 2004), and were observed with a PLAPLON 60X N.A. 1.42 objective. Images were acquired using FLUOVIEW FV1000 4.0.2.9 software and analyzed with Fiji Image J version 2.1.0/1.53c software. For FRAP (Fluorescence Recovery After Photobleaching) and FLIP (Fluorescence Loss in Photobleaching) analyses of the CSE-8-GFP strain, selective regions of interest (ROI) of the hyphae were selected for photobleaching and overexposed to 52% of the laser intensity for five seconds. Fluorescence intensity profiles at the SPK region during FRAP and FLIP experiments were analyzed in Fiji Image J using the image and batch tools in the Stowers plugins (https://research.stowers.org/imagejplugins/); easyFRAP web (https://easyfrap.vmnet.upatras.gr/) was also used for the analysis of a photobleached ROI in region II. In this case, a non-photobleached ROI in a distal zone of the hypha and an ROI in the background were used as controls. Spinning disk confocal microscopy (SDCM) was performed to observe the dynamics of CSE-8-GFP and CSE-7-mCherry. A Nikon ECLIPSE Ti-E Ti-E/B inverted microscope was used with a Yokogawa CSU-X1 confocal scanner unit, an ANDOR iXon Ultra camera, and an Apo 60X /0.13-0.21 oil immersion objective. Mean shift super-resolution (MSSR, Torres-García et al., 2022) analysis was applied to improve the resolution of the LSCM and SDCM images. We used an amplification parameter (AMP) value of 3 and a 0-order analysis, while the point spread function (PSF) value was obtained using the ‘ImageDecorrelationAnalysis’ plugin. Co-localization profiles were obtained with JACoP (Bolte and Cordelières, 2006).

### 2.4 Analysis of N-acetylglucosamine content in mutant strains

To further investigate the role of CSE-8 in the CHS secretory pathway and cell wall chitin synthesis, a colorimetric assay was performed to quantify GlcNAc content in the WT, *Δcse-8*, *Δcse-7*, and *Δcse-8; Δcse-7* strains following the specifications previously described (Morgan and Elson, 1934; Fajardo-Somera et al., 2015).

### 2.5 Bioinformatic analysis

To analyze the distribution of orthologues of CSE-7 (NCU05720) and CSE-8 (NCU01814) in the fungal kingdom and other organisms, an alignment of CSE-8 and CSE-7 was performed to identify the conserved domains of both proteins. The alignment was then used as input for HMMsearch (https://www.ebi.ac.uk/Tools/hmmer/search/phmmer) to obtain orthologous sequences to the CSE proteins. The resulting sequences were used for phylogenetic analysis, selecting amino acid sequences from representative species of each phylum. The sequences selected were aligned using MAFFT in Jalview v. 2.11.2.5. The resulting alignment was used to construct a phylogenetic tree by maximum likelihood using the Jones-Taylor-Thorton method, with a bootstrap value of 10000 and an amino acid substitution model. The phylogenetic tree obtained was edited in iTOL v6 (Letunic and Bork, 2024; https://itol.embl.de/tree/1589766147326311667424835#). The distribution of transmembrane domains, -sheets, and -helix structures displayed were carried out based on Uniprot (https://www.uniprot.org/) and AlphaFold (https://colab.research.google.com/github/sokrypton/ColabFold/blob/main/AlphaFold2.ipynb). Only structures predicted by AlphaFold with a pLDDT (per-residue measure of local confidence) greater than 50 were considered to outline the secondary structure of the proteins. Alpha-Fold and I-TASSER (https://zhanggroup.org/I-TASSER/) were utilized to predict the protein structures of CSE-8. Molecular docking analyses were performed using the HADOCK SERVER (Yan et al., 2020); http://hdock.phys.hust.edu.cn/) and AlphaFold 3 (Jumper et al., 2021; https://alphafoldserver.com). Predicted protein structures and potential interactions between CHS and CSE proteins were analyzed and visualized in PyMOL Molecular Graphics System, Version 2.5.4, Schrödinger, LLC.

### 2.6 Statistical analysis

All graphs and statistical analysis presented in this article were performed using GraphPad Prism version 9.

## 3 Results and discussion

### 3.1 CSE-8, a second *N. crassa* orthologue of the *S. cerevisiae* chitin synthase 3 (class IV) cargo receptor Chs7, is widely distributed throughout the fungal kingdom

Unlike in yeasts, little is known about the mechanisms governing the vesicular transport of CHSs in filamentous fungi. In order to investigate potential components of the protein machinery involved in this transport, the orthologues of the yeast Chs7 were identified in *N. crassa*. The function of the Chs7 orthologue CSE-7 (NCU05720) in the transport of CHS-4 (Class IV) has previously been described in *N. crassa* hyphae (Rico-Ramírez et al., 2018). CSE-7 acts as a cargo receptor for CHS-4, facilitating its transport from the ER to the SPK and septa. We identified a second Chs7 orthologue, NCU01814, and named it CSE-8 (for *chitin synthase export chaperone 8*) after confirming its role in CHS intracellular traffic.

**Figure 1:**
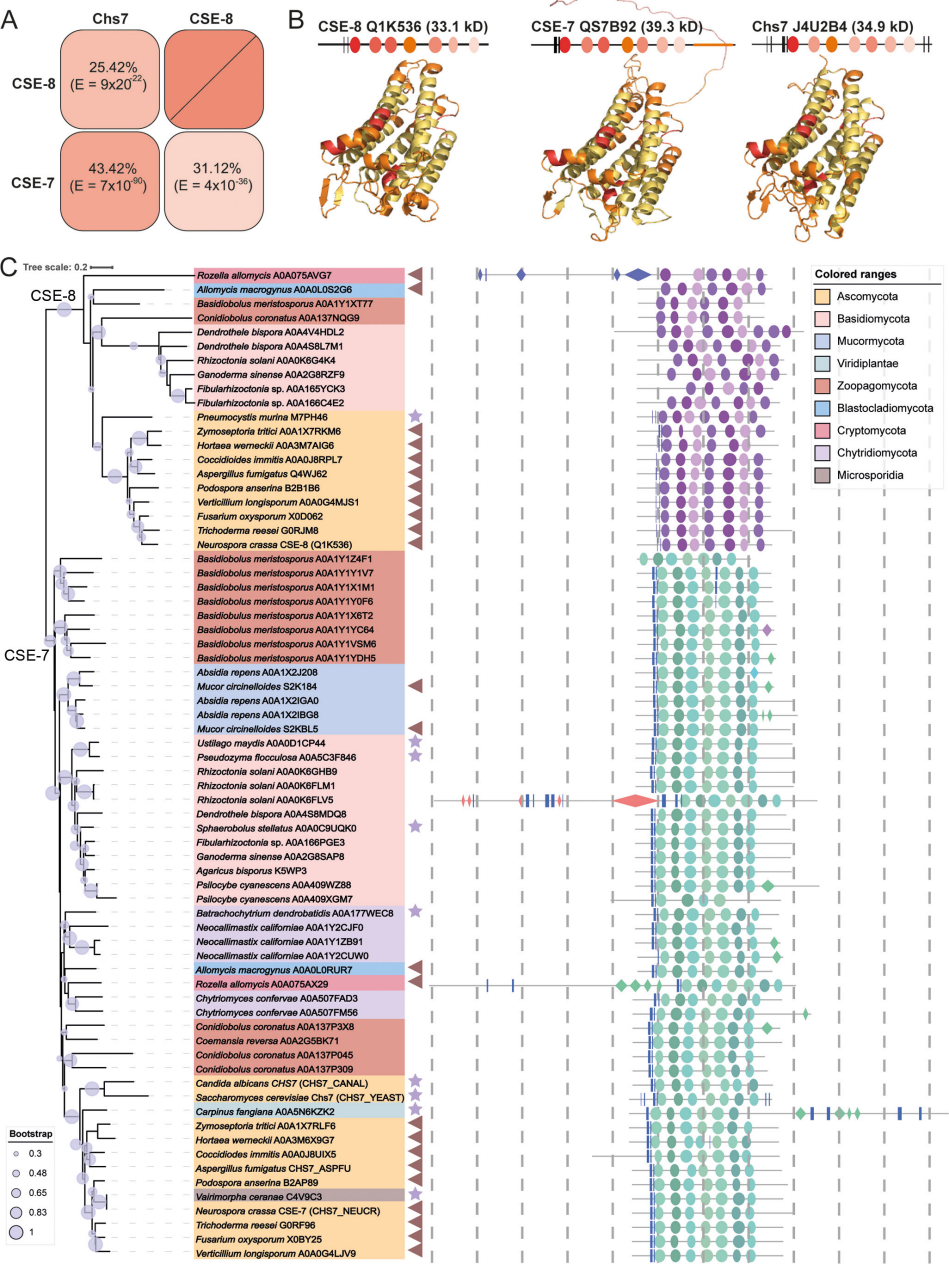
Protein secondary structure and phylogenetic distribution of CSE proteins. **(A)** Identity percent matrix of CSE-8 (NCU01814), CSE-7 (NCU05720) and Chs7 (YHR142W). **(B)** Secondary protein structures of CSE-7, CSE-8, and Chs7. Regions with the highest sequence conservation are colored in red, while transmembrane domains are shown in yellow. All three proteins feature seven transmembrane domains and two β-sheets. Notably, CSE-7 has a large, disordered region at its C-terminus. Models for these proteins were generated using AlphaFold 3, except for CHS-3, whose model was obtained from Swiss-Prot and aligned with the AlphaFold model. These structural models were used as the basis for molecular docking analyses. **(C)** Phylogenetic tree of Chs7 fungal orthologues. The phylogenetic tree includes representative species from each fungal phylum, showing that CSE proteins are highly conserved across all fungi. All identified proteins belong to the pfam12271 family, with only representative species displayed. Stars point to the species that present a unique CSE protein, and triangles indicate those species with two CSE copies. Bootstrap values are indicated by blue circles on each branch, with only values of 50% or greater considered reliable. The distribution of protein domains for each species is illustrated in schematic cartoons. Proposed secondary structures are based on AlphaFold models, with only regions having a pLDDT score greater than 50 displayed. The following nomenclature is used: rectangles for -sheets, diamonds for -helices, and ellipses for transmembrane domains. Each dotted line is equivalent to 100 aminoacids.

CSE-8 (Q1K536) was previously annotated as a hypothetical protein and classified as a member of the pfam12271 protein family, which includes proteins with the Chs3 catalytic domain. BLASTp analysis revealed that CSE-8 shares 25.42% identity with Chs7 (J4U2B4) and 31.12% with CSE-7 (Q7SB92; **Figure 1A**). Like CSE-7 (358 aa, 39.3 kDa) and Chs7 (316 aa, 34.9 kDa), CSE-8 (299 aa, 33.1 kDa) contains seven transmembrane alpha-helix regions and four cytoplasmic domains with conserved amino acid residues (**Figure 1B**). Despite their structural similarities, CSE-7 is unique in having a long-disordered domain at its C-terminal region (**Figure 1B**).

To assess the conservation of these proteins across the fungal kingdom, we performed an HMM search. These proteins had E-values ranging from 2.5 x 10^-152^ to 0.77. Of the identified sequences, 927 were from Ascomycota, 380 from Basidiomycota, 67 from Mucormycota, 45 from Zoopagomycota, 19 from Microsporidia, 26 from Chytridiomycota, 7 from Blastocladiomycota, and 2 from Cryptomycota. Representative species from each phylum were selected to construct the phylogenetic tree, where CSE proteins grouped into two distinct clades, one corresponding to CSE-7 orthologues and the other to CSE-8 orthologues. As expected, CSE-7 clustered with Chs7 from *S. cerevisiae*, reflecting the conserved identity between these two proteins. Most of the analyzed sequences grouped within the CSE-7 clade, which is characterized by the conservation of a disordered region in the C-terminal part of the protein. Most of the phylogenetically analyzed CSE proteins share common features, including two conserved β-sheets in the N-terminal region and seven predicted transmembrane domains. Most CSE proteins have a molecular weight below 45 kDa, fitting the typical profile of chaperones, which are usually small proteins. Larger CSEs (*Rozella allomycis* A0A075AX29 and A0A075AVG7, *Rhizoctonia solani* A0A0K6FLV5, *Carpinus fangiana* A0A5N6KZK2), are enriched with repeated β-sheet and -helix structures.

Different species had varying numbers of non-redundant sequences containing the characteristic domains of CSE-7 and CSE-8. For example, *Basidiobolus meristosporus* (Zoopagomycota), *Fibularhizoctonia* sp. (Basidiomycota), *Conidiobulus coronatus* (Zoopagomycota), and *Absidia rapens* (Mucormycota) have 9, 3, 4, and 3 proteins from the pfam12271 family, respectively (**Figure 1B**). Within the Ascomycota, filamentous species such as *Aspergillus* spp., *Penicillium* spp., *Trichoderma* spp., and *Fusarium* spp. have two genes encoding CSE proteins. Yeast-like and dimorphic species, such as *S. cerevisiae* and *C. albicans*, have only one CSE protein distributed in the CSE-7 clade. Only the single CSE of *Pneumocystis murina* was distributed in the CSE-8 clade. However, the dimorphic fungal pathogen *Coccidioides immitis* is an exception, possessing two CSE proteins. Another exception was *Ustilago maydis*, which retains a single copy of the CSE and has three morphological stages during its life cycle, including its yeast-like form (Cabrera-Ponce et al., 2012).

These results suggest that filamentous ascomycetous likely share a conserved mechanism for CHS function, with CSE-7 and CSE-8 serving as auxiliary proteins for the biogenesis of CHS vesicular carriers (**Figure 1**). Interestingly, one of the sequences identified in the alignment corresponded to a CSE protein from *Carpinus fangiana* (Viridiplantae, Streptophyta, Betulaceae), commonly known as Fang’s hornbeam. The homology of this sequence was confirmed by E-value (6.2 x 10^-118^) and bit score (405). The *C. fangiana* CSE protein (A0A5N6KZK2) is 709 amino acids long, contains six transmembrane regions, and is predicted to be a multipass membrane protein. Notably, this protein also preserves a homologous region with the α/β hydrolase fold superfamily (IPR029058), a diverse group of hydrolytic enzymes with different phylogenetic origins and catalytic functions. Additionally, it contains a pleckstrin homology domain, which is typically involved in protein targeting and signal transduction pathways. However, no experimental data currently exists about this unique plant CSE.

### 3.2 CSE-8-GFP localizes at sites of polarized growth and septa

**Figure 2:**
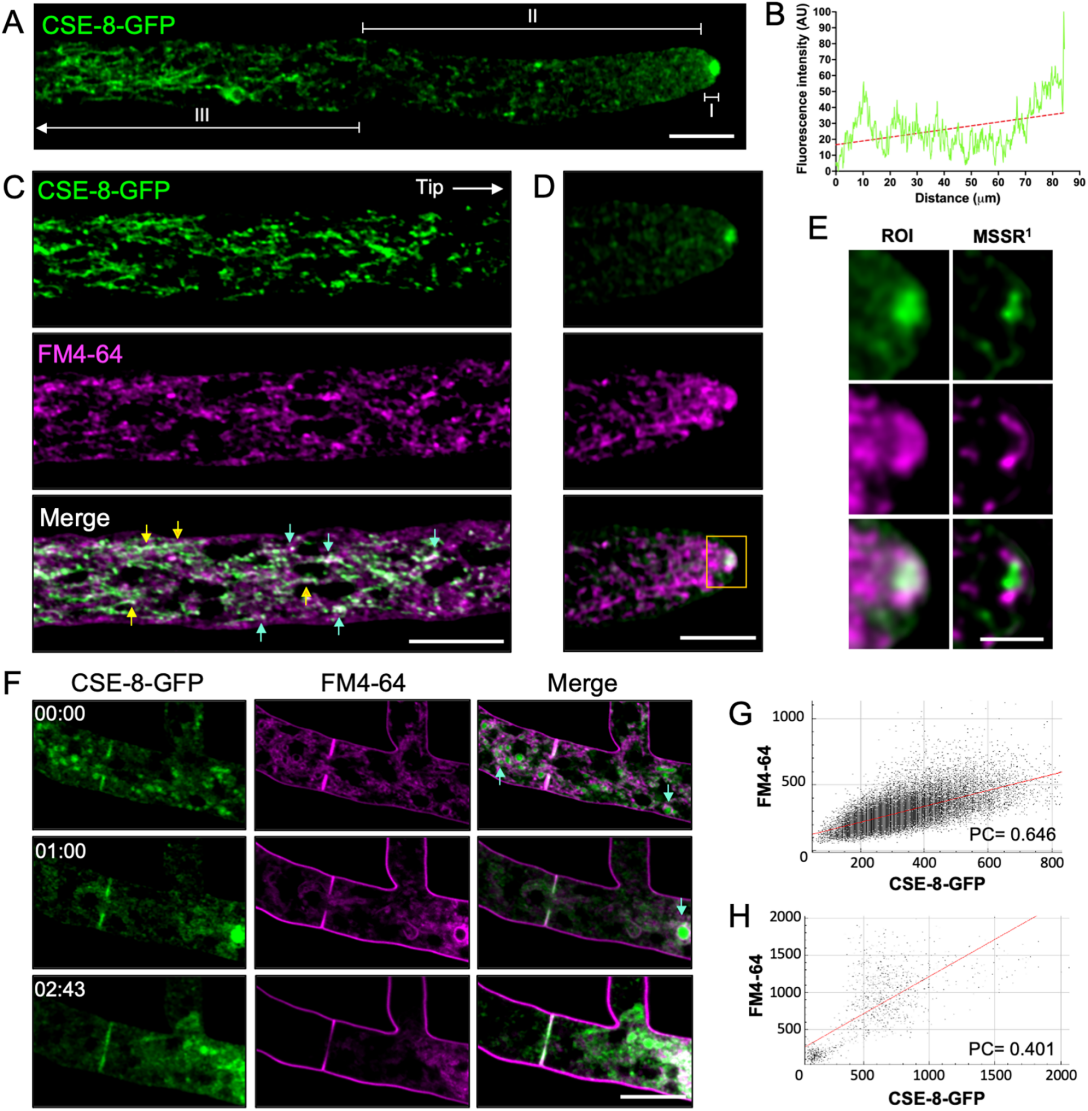
Subcellular localization of CSE-8 in *N. crassa* hyphae. **(A)** Distribution of CSE-8-GFP fluorescence in a hypha in the regions I, II, and III. **(B)** Graph of the Fluorescence intensity (FI) values along the hyphae displayed in **(A)**. **(C)** Distribution of CSE-8-GFP (green) and FM4-64 (magenta) in subapical regions; yellow arrows and blue arrows point to CSE-8-GFP in tubular compartments and vesicular clusters, respectively. **(D)** The apical region’s micrograph shows CSE-8-GFP localization at the SPK in hyphae stained with FM4-64 (channels are the same displayed in **(B)**. The yellow square shows the region of interest (ROI) shown in **(E)**. **(E)** Staining with FM4-64 (magenta) shows that CSE-8-GFP (green) accumulates at the SPK core using MSSR (analyzed using 1^st^ order equation). **(F)** Time series of CSE-8-GFP during septum formation, with FM4-64 marking the cell division sites. Blue arrows indicate CSE-8 in the lumen of globular compartments, where the membranes are stained with FM4-64. **(G)** Co-localization plot of CSE-8 with FM4-64 in the subapical region shown in **(B, H)** Co-localization plot of merged channels for the apical zone of the hyphae shown in **(D)** Note that the PC and plot confirms that CSE-8-GFP does not colocalize with the SPK’s outer layer stained with FM4-64. PC, Pearson’s coefficient; AU, Arbitrary units; scale bars = 10 μm, except for (E)= 2 μm.

To determine the subcellular localization of CSE-8 during polarized growth, the *N. crassa* homokaryon strain expressing CSE-8-GFP was analyzed using LSCM. The subcellular localization pattern of CSE-8-GFP was consistent with previous observations of CHS-tagged fluorescent proteins of *N. crassa* (Riquelme et al., 2007; Sánchez-León et al., 2011; Fajardo-Somera et al., 2015). There was a prevalence of CSE-8-GFP fluorescence in hyphal regions I and III, more precisely in the SPK and apical regions, as well as in subapical tubular structures (**Figures 2A–C**). FM4-64 staining was used to visualize the localization of CSE-8-GFP within the SPK region, revealing that CSE-8-GFP is concentrated at the SPK core, as shown more clearly through MSSR analysis (**Figures 2D, E**; **Supplementary Video 1**). CSE-8-GFP was also observed at septa in mature hyphae, co-localizing with FM4-64 mainly in the central region of the septum (**Figure 2F**). Additionally, CSE-8 shows partial overlap with FM4-64 in subapical regions of the hyphae, as confirmed by the co-localization plots (**Figures 2G, H**), indicating its involvement in endocytic pathways, particularly in vacuoles (Fischer‐Parton et al., 2000; Hickey et al., 2002). CSE-8-GFP’s appearance in FM4-64-stained organelles is consistent with studies on CHS recycling through the trans-Golgi network in a clathrin-dependent manner, as seen in *S. cerevisiae*, *C. albicans*, and *A. nidulans* (Seaman, 2008; Starr et al., 2012; Sacristan et al., 2013; Hernández-González et al., 2018; Knafler et al., 2019; Jin et al., 2021). Further research on CSE proteins will help clarify whether their recycling is tied to their association with CHS or an unrelated mechanism. These findings support a function of CSE-8 associated with CHS subcellular transport.

### 3.3 CSE-8 localizes to the endoplasmic reticulum

**Figure 3:**
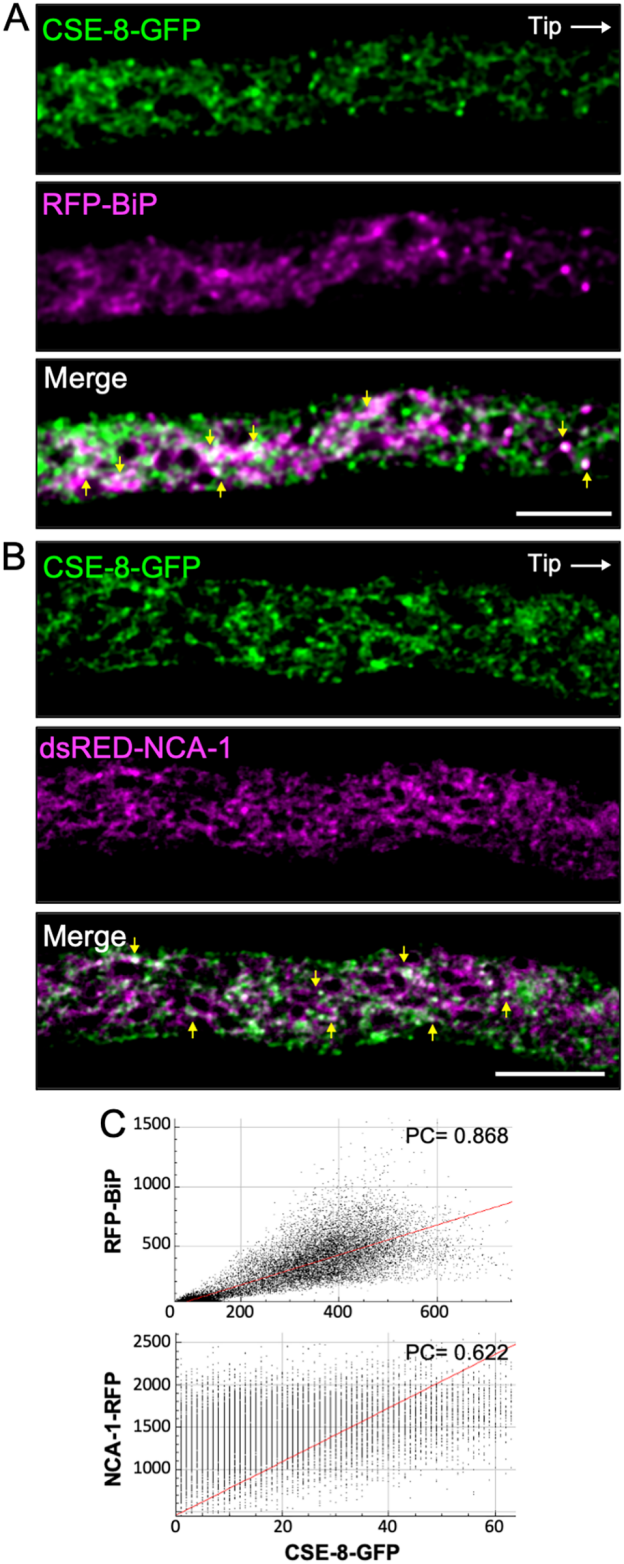
CSE-8-GFP and ER markers in heterokaryon strains of *N. crassa*. **(A)** Subapical region (20 μm from the tip) of a hypha co-expressing CSE-8-GFP and the ER lumen chaperone protein RFP-BiP. **(B)** Subapical region (30 μm from the tip) of a hypha co-expressing CSE-8-GFP and NCA-1-RFP, an ER SERCA-type Ca^2+^ ATPase. **(C)** Co-localization plots of the merged channels. The Pearsons’ coefficients indicate stronger co-localization between RFP-BiP and CSE-8-GFP than NCA-1-RFP and CSE-8-GFP. Scale bars = 10 μm.

We aimed to determine whether CSE-8 localizes to the ER, as its orthologue in *S. cerevisiae* (Chs7) has been identified as an ER chaperone, and the *N. crassa* CSE-7 was identified in the nuclear periphery (Trilla et al., 1999; Dharwada et al., 2018; Rico-Ramírez et al., 2018). We used RFP-BiP and dsRED-NCA-1 as markers of the rough ER and nuclear envelope, respectively. NCA-1, a homolog of the SERCA-type Ca^2+^-ATPase found in animal cells, primarily localizes to the nuclear envelope in *N. crassa* (Bowman et al., 2011). On the other hand, BiP, an HSP70 family protein, functions as an ER chaperone involved in post-transcriptional regulation, protein folding, and the recognition of misfolded proteins destined for the unfolded protein response (UPR) pathway under ER stress (Hendershot et al., 1995). For this work, we renamed the *N. crassa* GRP-78 protein as BiP (Monnerjahn et al., 2001; Bowman et al., 2011) based on its orthology to the mammalian and plant binding immunoglobulin protein and the yeast Kar2. The co-localization of CSE-8-GFP with dsRED-NCA-1 or RFP-BiP in heterokaryon strains was observed in region III of the hyphae (**Figures 3A, B**). Co-localization analysis confirmed the presence of CSE-8 at ER membranes and, to a lower degree, around the nuclei (**Figure 3C**). Pearson’s coefficients showed a stronger correlation between BiP and CSE-8 than between NCA-1 and CSE-8 (**Figure 3C**), further suggesting that CSE-8 is an ER protein potentially involved in the biogenesis of CHS-carrying microvesicles from the ER, as previously reported for CSE-7 and Chs7.

### 3.4 Apical CSE-8 arises from subapical regions of the hyphae

**Figure 4:**
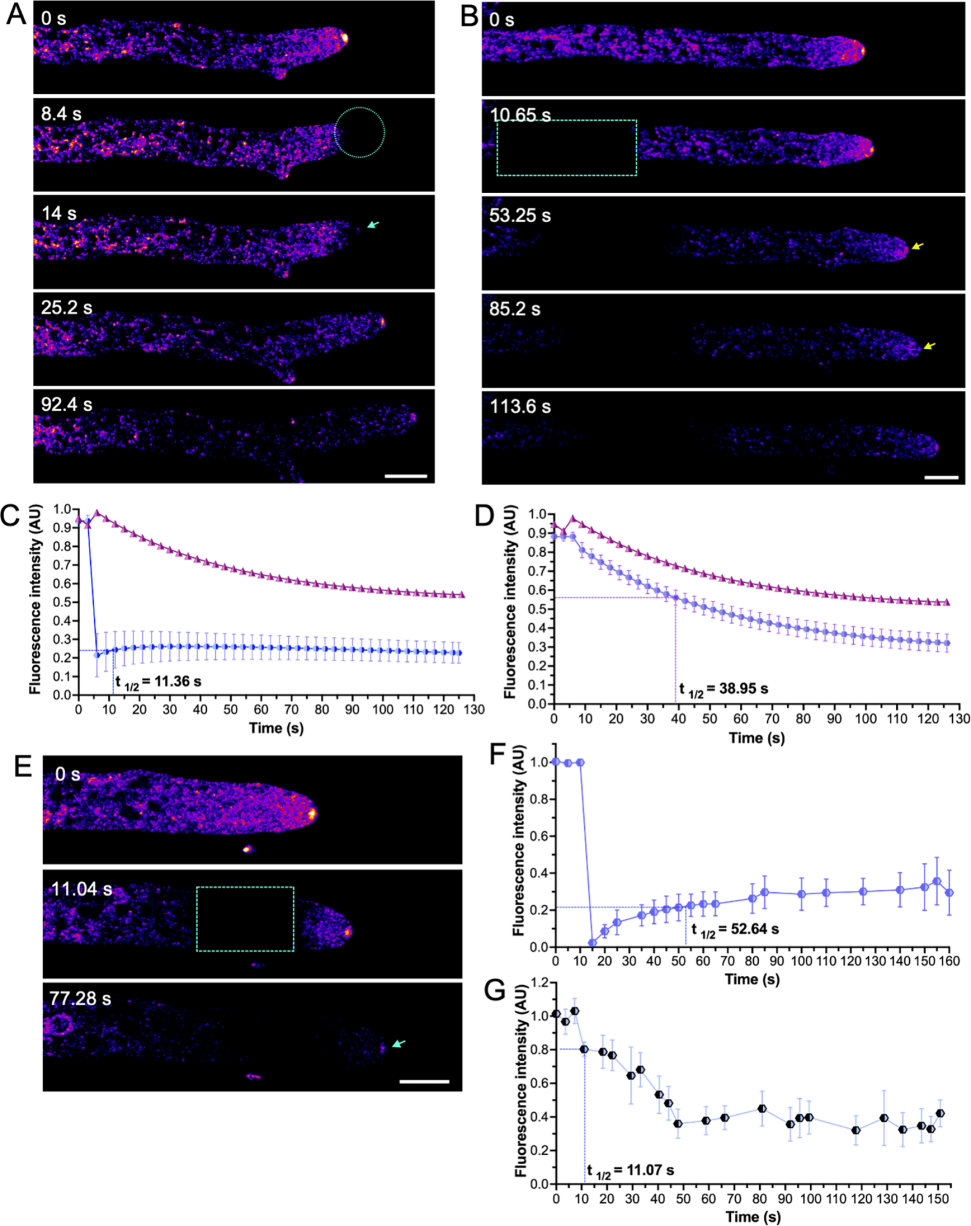
Fluorescence Recovery After Photobleaching (FRAP) and Fluorescence Loss in Photobleaching (FLIP) of CSE-8-GFP. **(A)** FRAP at the SPK region. The photobleached area is indicated by a cyan circle, with the blue arrow in the third panel marking the re-establishment of CSE-8-GFP fluorescence at the SPK. **(B)** Time-lapse of subapical FRAP of CSE-8-GFP. The cyan box highlights the photobleached region, and the blue arrow indicates the fluorescence loss at the SPK after photobleaching. Fluorescence intensity at the SPK (FLIP) following photobleaching at apical **(C)** and subapical regions **(D)**. The measured area in **(A)** corresponds to 5.83 px^2^ in the SPK region, while the measured area in **(B)** covers 22.8 px^2^. **(E)** Time series of the photobleached region II during FRAP experiments. **(F)** Fluorescence intensity profiles of the photobleached region II indicated in the cyan box (measured area of 12.44 px^2^). **(G)** Fluorescence intensity profiles in the SPK (FLIP) during FRAP are shown in Figure E (measured area of 4.32 px^2^). The bars in the plots indicate the standard error of the mean calculated for each time point (n = 4). In both graphs, the fluorescence intensity at the SPK in non-photobleached hyphae corresponds to the pink line (control). The 50% fluorescence recovery time (t_1/2_) is shown on each graph. Notably, fluorescence is more affected throughout the experiment when photobleaching occurs in the subapical region than in the SPK. Yellow and cyan arrows indicate CSE-8-GFP at the SPK. For FLIP experiments shown in B, all selected ROIs for bleaching were positioned 60 ± 5 μm from the tip. AU: arbitrary units; scale bars = 10 μm.

FRAP and FLIP experiments were carried out to elucidate the biogenesis of CSE-8 in *N. crassa* hyphae (**Figure 4**). We first assessed whether photobleaching the apex (within the first 5.63 ± 2.18 μm from the tip) affected the fluorescence of CSE-8-GFP at the SPK, where it accumulates, presumably co-transporting CHS. After photobleaching the SPK region, CSE-8-GFP fluorescence at the SPK recovered about 6.06 ± 0.34 s after photobleaching (**Figure 4A**). Since CSE-8-GFP displayed high fluorescence intensity at distal regions of the hypha (**Figures 2A, B**), we also photobleached a distal ROI (67.33 ± 6.38 μm from the tip) and measured fluorescence intensity at the SPK (**Figure 4B**). Fluorescence loss after photobleaching in the subapical region was observed, including a notable decrease in CSE-8-GFP fluorescence at the SPK. The half-time fluorescence recovery value at the SPK was greater for FLIP when applying the photobleaching at an ROI in region III of the hyphae (t_1/2_ = 38.95 s), than for FRAP applied directly at the SPK (t_1/2_ = 11.36 s) (**Figures 4C, D**). To support our observations, the photobleaching was also applied to region II of the hyphae (10.99 ± 0.78 μm from the apex), which is characterized by a high abundance of nuclei (**Figure 4E**). FRAP analysis displayed a t_1/2_ value of 52.64 s for the photobleached ROI (**Figure 4F**). Fluorescence at the SPK did not completely disappear following photobleaching at region II, suggesting that most CSE-8-GFP vesicles originate from more distal regions (**Figure 4G**), rich in rough ER (Martínez-Andrade et al., 2024). Additionally, a significant portion of the fluorescence may derive from the network of endomembranous cisternae (NEC), where CSE-7 has been previously localized (Rico-Ramírez et al., 2018). These findings suggest that CHS synthesis occurs in the subapical regions and that efficient transport to the apex is essential for polarized growth.

### 3.5 CSE-8 and CSE-7 travel in different vesicles in *N. crassa* hyphae

**Figure 5:**
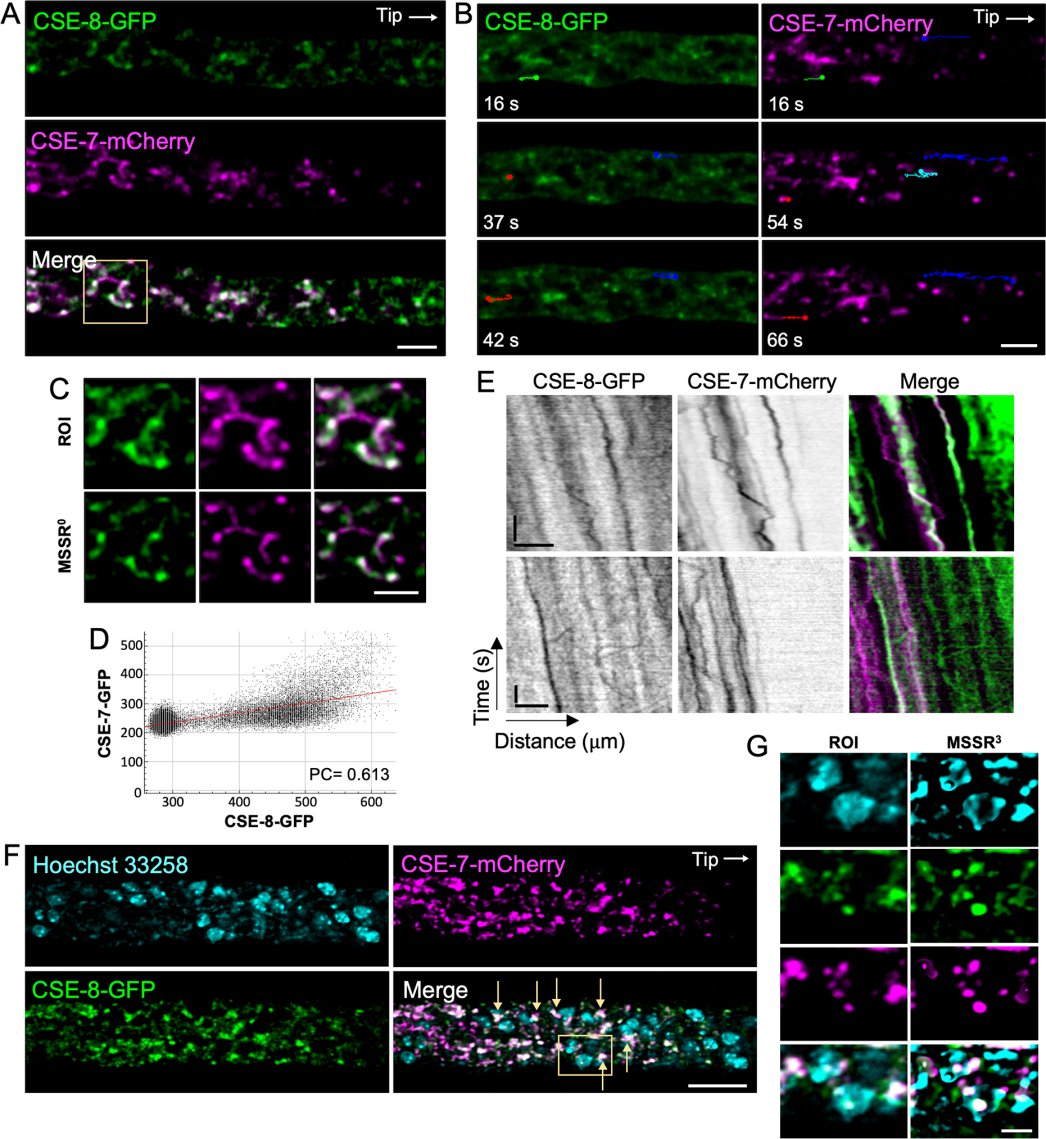
Spinning disk confocal microscopy revealed the movement of CSE-7-mCherry and CSE-8-GFP in different vesicles. **(A)** Subapical hyphal region (20 μm from the tip) of an *N. crassa* heterokaryon strain co-expressing CSE-7-mCherry and CSE-8-GFP. White points indicate areas of overlap between the two channels. **(B)** Tracking of anterograde and retrograde movement of CSE-7-mCherry and CSE-8-GFP clusters in the subapical region of the hyphae. Color lines show the trajectories of particular vesicle clusters. Green and red trajectories indicate anterograde movements. Blue and cyan indicate retrograde trajectories followed by anterograde movements. Points at the end of the lines indicate the final position of the clusters for each frame. **(C)** The selected ROI with CSE-8-GFP and CSE-7-mCherry around unidentified organelles shows partial overlapping signals for both CSE proteins. The ROI was analyzed using the zero-order equation of the MSSR algorithm (analyzed using zero-order equation). **(D)** Co-localization analysis of the merged image shown in **(C)**. **(E)** Kymographs of CSE-7-mCherry and CSE-8-GFP; scale bars correspond to 10 μm (x axis) and 10 s (y axis). **(F)** LSCM imaging of the heterokaryon strain shown in panels A and B with stained nuclei (cyan). Yellow arrows point to overlap of the CSE-8-GFP and CSE-7-mCherry signals around the nuclei. **(G)** ROI selected in the merge channel of panel F, analyzed using the MSSR plugin in Fiji; a third-order equation was selected for analysis. Scale bars: **(A, B, D–F)** = 10 μm, **(C–G)** = 2 μm.

CSE-8 exhibits a subcellular distribution similar to the one previously reported for CSE-7 by Rico-Ramírez et al. (2018). To compare the dynamics of these two proteins, a heterokaryon strain co-expressing CSE-8-GFP and CSE-7-mCherry was analyzed using SDCM. We used SDCM rather than LSCM in order to observe the dynamics of the two proteins in near real-time and to avoid possible spurious co-localization due to scanning time. When the two fluorescence channels were superimposed, overlapping spots of CSE-8 and CSE-7 were observed at subapical regions of the hyphae (**Figure 5A**; **Supplementary Video 2**), around non-fluorescent round organelles. The MSSR plugin in Fiji (Torres-García et al., 2022) was used to enhance the resolution of the confocal images, confirming a partial co-localization between CSE-8 and CSE-7 (**Figures 5C, D**). In addition, microscopy revealed that some GFP and mCherry vesicle clusters moved independently in both retrograde and anterograde directions (**Figure 5B**). In the kymographs corresponding to the subapical region of the hyphae, it can be seen in more detail that both CSE-8-GFP and CSE-7-mCherry have independent trajectories mainly in the anterograde direction, although retrograde displacements are also observed (**Figure 5E**). These results reveal that CSE-8 and CSE-7, are transported in different vesicle sub-populations. To determine whether the organelles observed near CSE-7 and CSE-8 were nuclei (**Figure 5B**), the nucleic acid dye Hoechst 22358 was used (**Figure 5F**). MSSR revealed clusters of GFP and clusters of mCherry around the nuclei, with some clusters partially overlapping (**Figure 5G**). Remarkably, non-fluorescent organelles surrounded by CSE-7-mCherry and CSE-8-GFP are still observed in the hypha, suggesting that there could be compartments, other than nuclei, related to both proteins.

### 3.6 Lack of CSE-8 prevents CHS-3-GFP from reaching the SPK and septa

**Figure 6:**
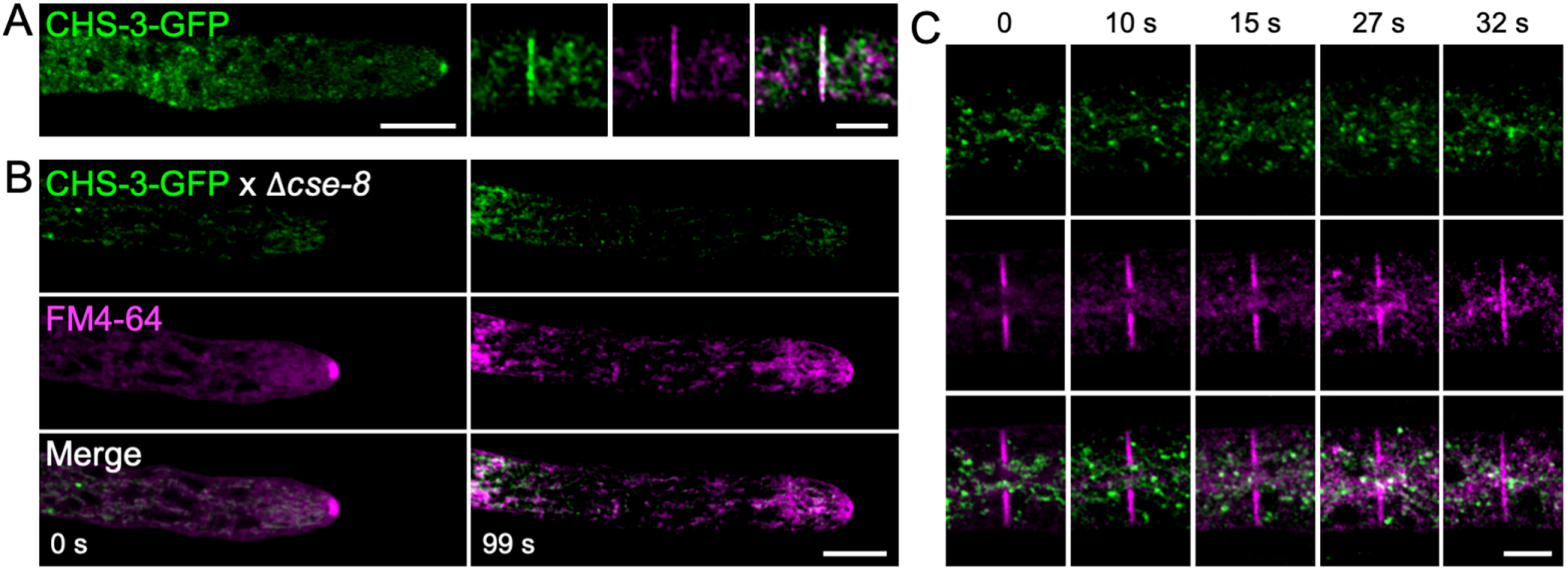
Lack of *cse-8* prevents CHS-3-GFP from reaching sites of cell wall biosynthesis. **(A)** Laser scanning confocal microscopy of the *N. crassa* strain with exogenous labeling of CHS-3-GFP shows CHS-3-GFP clearly visible in the SPK and in FM4-64-stained septa. **(B)** In the *Δcse-8* strain expressing CHS-3-GFP, hyphal staining with FM4-64 reveals the absence of CHS-3 in the SPK core at 99 seconds, where the outer layer is still visible via FM4-64 staining. **(C)** Time series showing disrupted CHS-3-GFP transport in the *Δcse-8* strain, using FM4-64 as a marker for SPK and septum formation. The time-lapse of septum formation in the *Δcse-8* knockout strain expressing CHS-3-GFP shows that the fluorescence of FM4-64 in the forming septum does not overlap with the surrounding CHS-3-GFP vesicle clusters. Scale bars = 10 μm.

Genetic crosses were performed between a strain expressing CHS-3-GFP (NSSG6) (**Figure 6A**) and a *Δcse-8* knockout strain to investigate potential interactions between CSE-8 and CHS in *N. crassa*. Homokaryotic strains recovered from the resulting ascospores were analyzed by LSCM to confirm the presence of fluorescence, followed by validation through PCR using primers flanking the CHS-3-GFP and *cse-8* gene constructs (**Supplementary Figure 1**). In homokaryotic *Δcse-8* strains expressing CHS-3-GFP, fluorescence appeared in subapical clusters, with an absence of signal at the SPK and septa (**Figures 6B, C**). A previous study including FRAP analysis of CHS-3-GFP and CHS-6-GFP revealed that photobleaching near the hyphal tip caused only transient disruption in CHS localization at the SPK (Riquelme et al., 2007). Furthermore, the localization of CSE-8-GFP within the lumen of globular vacuoles is consistent with earlier findings for CHS-3-GFP (Riquelme et al., 2007). These results support the hypothesis of intracellular transport of CSE-8 in association with CHS-3. Additionally, *Δcse-7* and *Δcse-8* strains expressing CHS-1-GFP or CHS-5-GFP were obtained (**Supplementary Figure 2**). Since no disruption in CHS transport to polarized growth sites was observed for CHS-1 (class III) or CHS-5 (class V), which are unique to filamentous fungi, it is suggested that these CHS may employ alternative transport routes, bypassing ER-to-Golgi COPII vesicles, as proposed in previous studies (Riquelme et al., 2007; Sánchez-León et al., 2011; Rico-Ramírez et al., 2018). Thus, while CSE proteins may still function as chaperones assisting with CHS folding at the ER, they do not appear to be essential for CHS transport to apical regions in filamentous fungi, suggesting the evolution of alternative transport mechanisms.

To explore the possible interaction mechanism between CHS-3 and CSE-8, AlphaFold 3 and the HDOCK server were used to predict potential interaction sites. The model with the lowest average distance values between the atoms was selected and compared to the docking results obtained from AlphaFold 3, which yielded a pTM score of 0.68. A CHS-3 dimer obtained from SwissProt was used for the interaction models with CSE-8 to better predict their real interaction (**Supplementary Figure 3A**). This model of the *N. crassa* CHS-3 dimer is supported by crystallographic data on CHS structures from *S. cerevisiae* and *C. albicans* (Chen et al., 2022; Ren et al., 2022; Chen et al., 2023) and is consistent with the structure of Chs1 (class I) from the oomycete *Phytophthora sojae* and Chs2 (class I) from *Candida albicans* (Chen et al., 2022; Ren et al., 2022). In order to obtain more accurate results, the models displayed in **Supplementary Figure 4** were used as controls.

According to the interaction model, the 5th, 6th, and 7th transmembrane domains of CSE-8 interact with transmembrane regions 1, 2, 6, and 7 of one CHS-3 unit (**Supplementary Figures 3B, C**). The most stable *in silico* interaction was predicted between the beta-sheet LPLC domain of CSE-8 and the charged amino acids glutamate, aspartate, and arginine located at the C-terminal end of CHS-3 (**Supplementary Figures 3C, D**). Additionally, potential interactions between a pair of hydrophobic amino acids in the second unit of the CHS-3 dimer were suggested (**Supplementary Figure 3D**). This interaction model aligns with experimental results on the Shr3 chaperone, whose transmembrane domains protect charged amino acids on the N-terminal end of the amino acid permease Gap1 (Kota et al., 2007). Interestingly, the *in silico* interaction between the β-folded structures of CSE-8 and the transmembrane domains of CHS-3 mirrors behaviors observed in other chaperones. These proteins use their β-sheet structures to facilitate protein folding, prevent accumulation, and contribute to protein oligomerization (Sun and MacRae, 2005; Karamanos et al., 2020).

### 3.7 CSE-8 and CHS-3 transport under ER stress conditions

**Figure 7:**
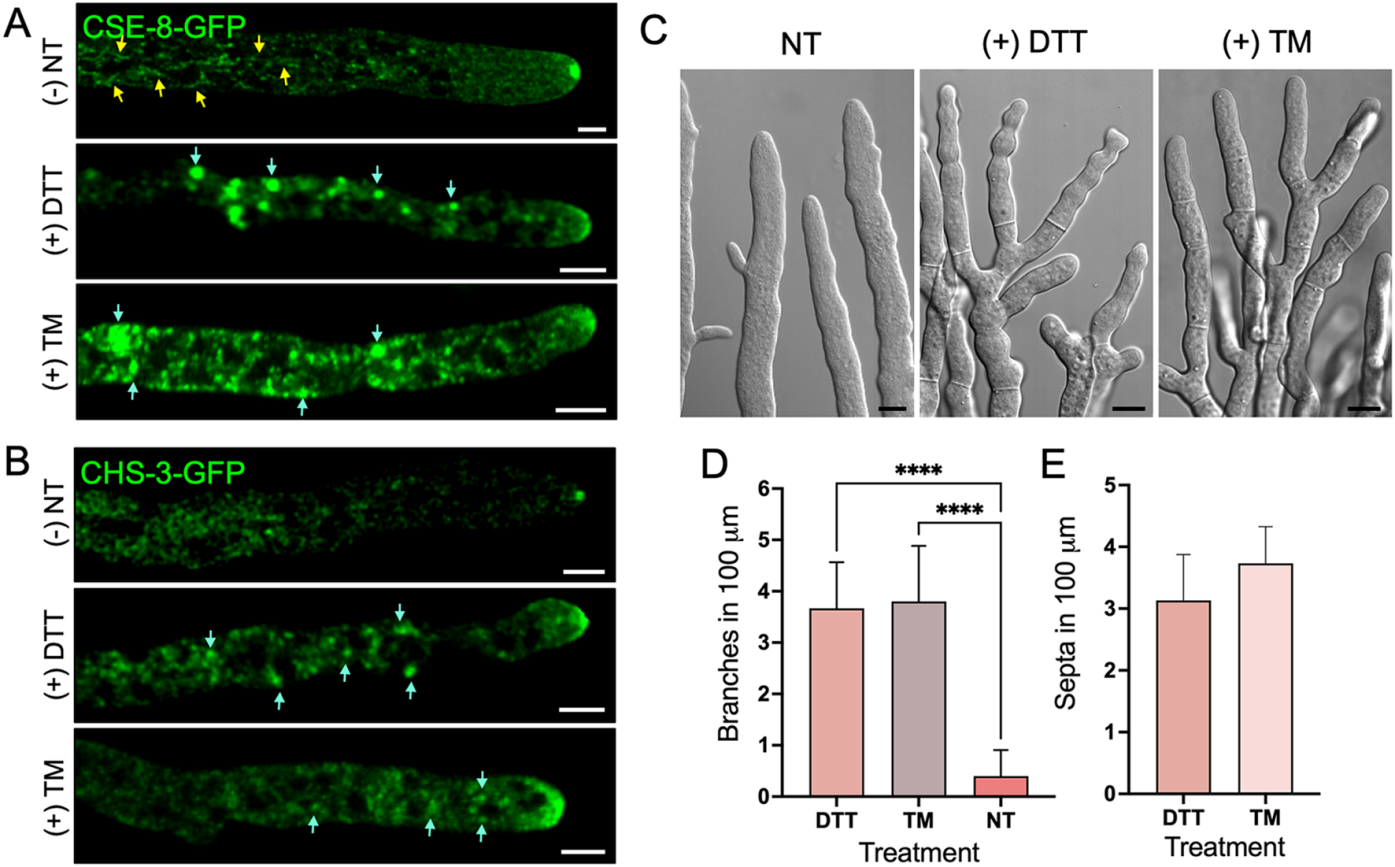
ER stress induced by DTT and TM stressors affects the growth and cell polarity of *N. crassa*. **(A)** Micrographs of the CSE-8-GFP tagged strain exposed to ER stress treatments with DTT (1.25 mM) and TM (4.25 μg/mL). Yellow arrows point to CSE-8-GFP in subapical tubular endomembranes before ER stress treatments, while blue arrows point to the accumulation of CSE-8-GFP in larger globular bodies after treatments. **(B)** Micrographs of the strain expressing CHS-3-GFP before and after ER stress treatment. Blue arrows indicate the accumulation of CHS-3-GFP along the hyphae. **(C)** DIC micrographs of hyphae treated with DTT and TM showing the presence of septa near apical regions and hyperbranched hyphae. **(D, E)** Quantification of branches and septa within the first 100 μm of principal hyphae treated with DTT and TM (n=15 for each treatment); NT, non-treated hypha). Due to the absence of septa in the first 100 μm from the tip in hyphae of the WT strain, no values are shown in graph E for NT hyphae. (****) indicate significant differences for quantified hyphal branching with and without treatment (p-value of 0.0001). PC, Pearson’s coefficient; scale bars = **(A, B)** 5 μm; **(C)** = 10 μm.

To further investigate the role of CSE-8 in CHS-3 transport, we examined their subcellular transport under conditions of ER stress induced by DTT and TM (**Figure 7**). The minimum inhibitory concentrations for observing the effects of these stressors on protein transport were determined to be 1.25 mM for DTT and 4.25 μm/mL for TM. As a positive control for ER stress, the *N. crassa* heterokaryon strain expressing CSE-8-GFP and RFP-BiP was grown (**Supplementary Figures 5A, B**). TM produced a similar effect to DTT on RFP-BiP distribution (**Supplementary Figures 5E, F**). In subapical regions near the tip, globular clusters of both proteins were observed, indicating that CSE-8-GFP is retained in the ER under stress conditions (**Figures 7A, B**). RFP-BiP, an established ER stress reporter, accumulated in subapical globular bodies in hyphae treated with DTT or TM, consistent with Kar2-sfGFP localization in stressed yeast cells (Lajoie et al., 2012). DTT also produced similar effects in hyphae expressing CSE-7-GFP or CHS-4-GFP, supporting the notion that CSE proteins play a comparable role in vesicular trafficking from the ER (**Supplementary Figure 6**). Likewise, CHS-3-GFP accumulated in subapical regions under DTT or TM stress, with complete SPK disruption observed at the hyphal tip. CHS-3-GFP appeared as an apical vesicle crescent and in smaller clusters than those presented by CSE-8-GFP (**Figure 7B**).

Despite DTT exposure, CSE-8-GFP and CHS-3-GFP still reached the hyphal tip, forming a disorganized structure like an apical crescent at the apex. FM4-64 staining revealed complete disruption of the SPK, including its outer layer, which surrounds the core visualized by CSE-8-GFP in non-treated cells (**Supplementary Figures 5C, D**). TM did not severely disrupt the SPK, likely due to its primary effect on the folding of glycosylated proteins. It is noteworthy that, under ER stress, CSE-8-GFP predominantly appeared in globular structures resembling globular vacuoles along the hyphae. These globular vacuoles were more pronounced in the CSE-8-GFP strain than in the CHS-3-GFP strain. In contrast, CHS-3-GFP was observed in smaller clusters near the tip. These findings suggest two key conclusions: first, CSE-8’s presence in the NEC could indicate its localization within the ER of this network. Second, the significant accumulation of CSE-8-GFP in globular vacuoles along the hyphae may result from the activation of the unfolded protein response degradation or other autophagic pathways. Further studies are required to determine the exact pathways CSE-8 and CHS-3 follow under stress ER conditions. However, these experiments confirm that CSE-8 is an ER protein based on its subcellular distribution in response to stress.

Severe loss of polarity, hyperbranching, and excessive septa formation in DTT- and TM-treated hyphae underscore the importance of ER integrity for polarized growth (**Figures 7C, E**). CSE-8 and CHS-3 transport appear essential for maintaining this growth, likely activated in response to ER stress to sustain essential cellular processes like chitin synthesis. The constant supply of *S. cerevisiae CHS7* under ER stress was identified among genes up-regulated under UPR cell conditions induced by TM and DTT (Travers et al., 2000; Weichet et al., 2020). In the same study, genes corresponding to exomer subunits Chs5 and Chs6 were also up-regulated in response to ER stress. These findings suggest a possible activation of *cse* genes and other selected genes in response to ER stress to support key developmental processes, including chitin synthesis. Further studies on the response of *cse-8* and *cse-7* genes under ER stress conditions may help explain the role of CSE proteins in CHS-3 and CHS-4 transport from the ER.

### 3.8 CSE-8 RE sorting occurs into COPII vesicles

The next step in elucidating the vesicular trafficking pathway of CHS-3, using CSE-8 as a chaperone, was to investigate whether CHS-3 could exit the ER and enter COPII vesicles. Brefeldin A (BFA) inhibits COPII vesicle formation and, consequently, the ER-to-Golgi vesicular transport pathway, leading to the disruption of Golgi cisternae and accumulation of coatomer subunits in ER transition membranes, forming BFA bodies (Orci et al., 1993).

**Figure 8:**
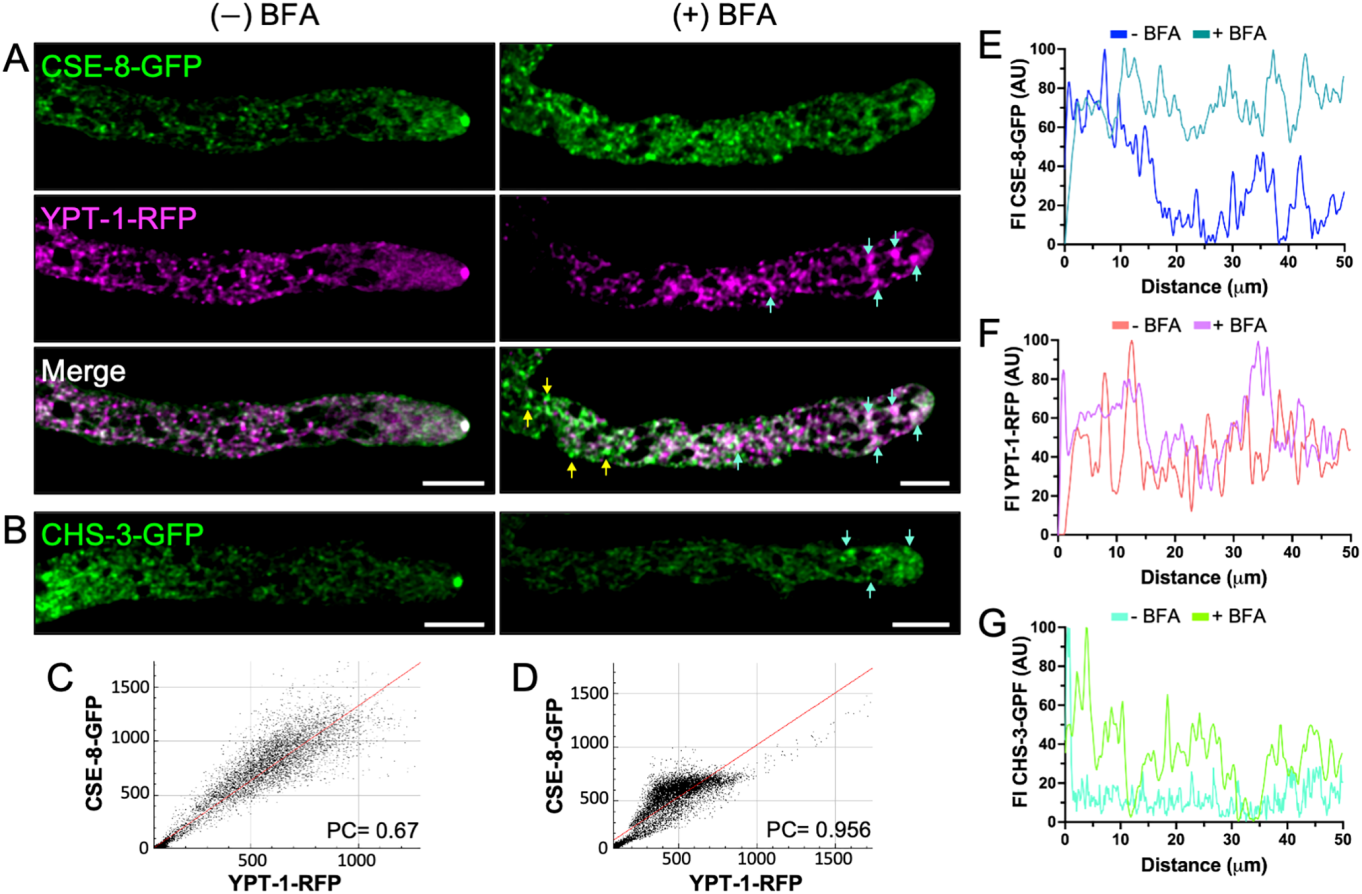
Brefeldin A disrupts the subcellular transport of CSE-8-GFP and CHS-3-GFP to the SPK. **(A)** Hypha co-expressing YPT-1-RFP and CSE-8-GFP without treatment (left) and exposed to BFA at 200 μg/mL (right). In the BFA-treated hyphae, CSE-8-GFP fails to reach the SPK and accumulates at the beginning of region II in putative BFA bodies labeled with YPT-1-RFP. Blue arrows indicate the accumulation of CSE-8-GFP in BFA bodies marked by YPT-1-RFP, while tallow arrows point the accumulation by itself. **(B)** Disruption of subcellular transport of CHS-3-GFP in hyphae treated with BFA. Blue arrows point the accumulation of CHS-3-GFP in regions near the tip. **(C, D)** Plots of the co-localization analysis shown in **A** (**C (-)** BFA; **D** (+) BFA). Pearsons’ coefficient is greater in BFA-treated samples, indicating that a subpopulation of CSE-8-GFP accumulates with YPT-1-RFP in putative BFA-bodies. **(E–G)** Fluorescence intensity plots for the first 60 μm of hyphae shown in **(A, B)**. Scale bars = 10 μm.

A strain expressing the YPT-1-RFP construct was used as a control and grown in VMM medium poisoned with BFA to ensure Golgi disruption. In this strain, YPT-1-RFP localized to the SPK and accumulated in globular bodies within the subapical region (**Figure 8**), a hallmark of Golgi membrane disruption. Similarly, CSE-8 accumulated in BFA-induced bodies along the hypha, with prominent accumulations into BFA bodies in the 1^st^ and 2^nd^ regions (**Figures 8A, C, D**). CHS-3-GFP clusters were also observed near the tip (**Figure 8B**). BFA treatment led to an increase in fluorescence intensity across all regions, confirming the disruption of CSE-8-GFP, YPT-1-RFP, and CHS-3-GFP transport (**Figures 8E-G**). These results indicate that CSE-8 is transported to the Golgi in COPII vesicles, following the canonical transport route to the hyphal tip. This is consistent with previous observations for Chs3, where the cargo receptor Erv14 is required for its exit from the ER in COPII vesicles (Sacristan et al., 2013).

### 3.9 The phenotypes of the *Δcse-8* and *Δcse-7 Δcse-8* strains confirm their role in chitin synthesis

**Figure 9:**
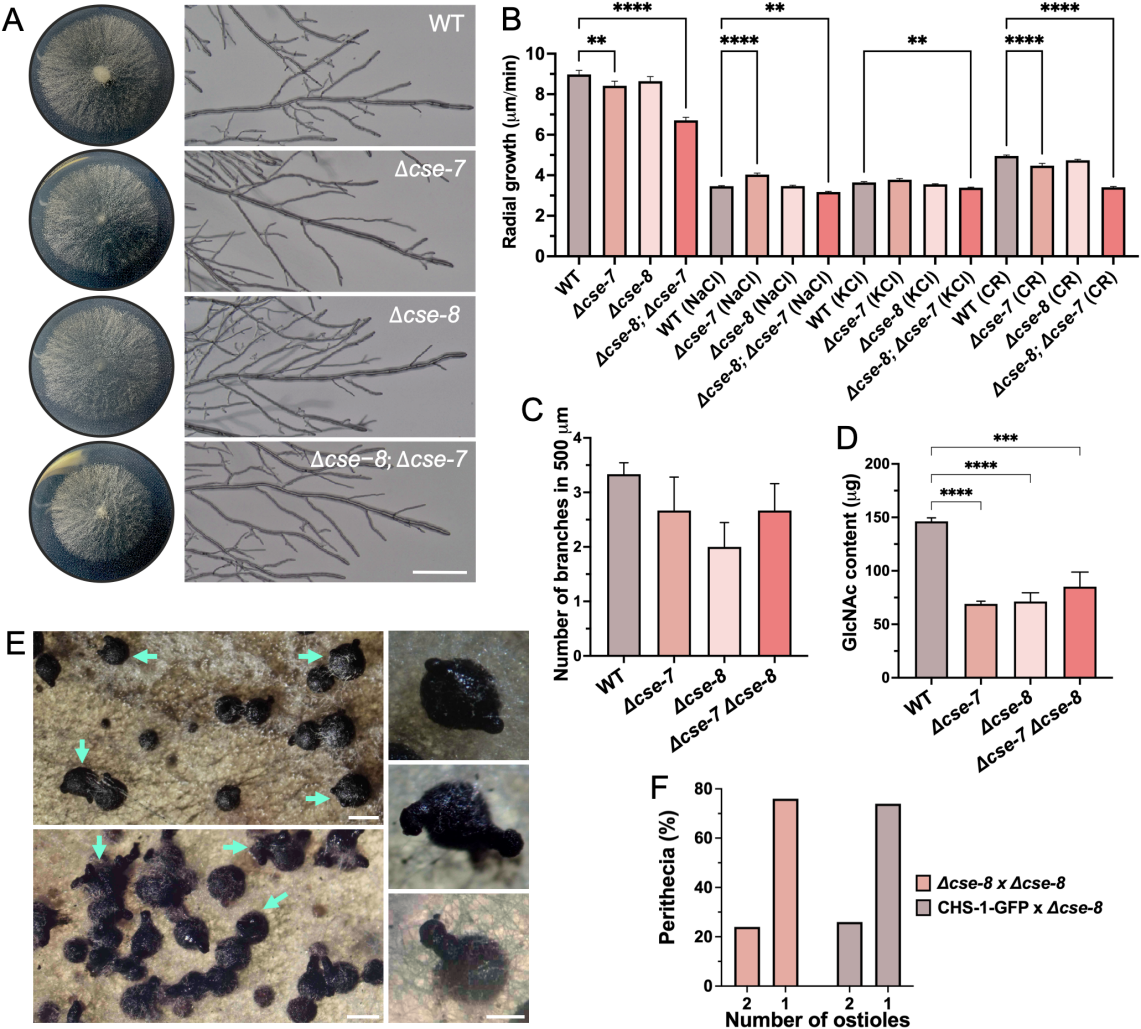
Phenotypic characterization of *N. crassa Δcse-7*, *Δcse-8*, and *Δcse-8; Δcse-7* strains. **(A)** Colony growth and hyphal morphology of the three strains compared to the WT strain (Scale bar = 250 μm). **(B)** Quantification of radial growth (n = 30) of all the strains exposed to osmotic (NaCl 0.8 mM; KCl 0.8 mM) and cell wall (Congo Red 100 mg/mL) stress conditions. **(C, D)** Graphs showing differences in branching (n = 10) and N-acetylglucosamine content (n = 4) of the analyzed strains. Asterisks indicate significant differences between strains, with p-values of 0.001 (**), 0.005 (***) and 0.0001 (****). **(E)** Perithecia from homozygous *Δcse-8* crosses after 15 days (top) and 30 days (bottom) of growth in SCM. Blue arrows denote perithecia, displaying the “shaka” phenotype (two ostioles). Perithecia with unique ostiole is shown in the bottom square. **(F)** Quantification of “shaka” perithecia produced *Δcse-8* sexual crosses (n= 100). *Δcse-8* x CHS-1 corresponds to perithecia obtained by crossing between *Δcse-8* knockout and FGSC #13408 strains (Scale bars= 200 μm).

To investigate the function of CSE proteins, we characterized corresponding mutants to identify any distinct phenotypic differences. No significant differences in colonial growth were observed in the *Δcse-8* and *Δcse-7* single mutants (**Figures 9A, B**). However, the *Δcse-8; Δcse-7* double mutant exhibited a slower growth rate than the single mutants and the WT strain (**Figures 9A, B**). Growth of the *Δcse-8* and *Δcse-7* mutants was significantly impaired under osmotic stress conditions induced by high concentrations of NaCl and KCl (**Figure 9B**). Furthermore, both the double mutant and *Δcse-7* were more sensitive to Congo red (CR)-induced stress than the WT. CR is a molecule known for its antifungal activity, as it can inhibit chitin synthesis and form complexes with chitin and glucans present in the cell wall (Bartnicki-Garcia et al., 1994; Ram and Klis, 2006). Taken together, these results indicate that the absence of *cse-7* and *cse-8* renders *N. crassa* hyphae more sensitive to osmotic stress-induced changes in turgor pressure, as well as to antifungal agents targeting cell wall and chitin synthesis, directly linking both genes to the mechanisms of chitin synthesis in cell wall. The average number of branches at a selected distance from the tip of the main hyphae was lower in the mutants tested (**Figure 9C**). However, statistical analyses did not reveal any significant difference in this morphological characteristic. Levels of the chitin monomer GlcNAc were consistent across all three mutants, each showing approximately 50% less GlcNAc than the WT strain (**Figure 9D**). Notably, knockout mutants *Δchs-5*, *Δchs-6*, *Δchs-7*, and RIP mutant *chs-1* — mutants in *chs* specific to filamentous fungi — display a severely affected radial growth phenotype (Yarden and Yanofsky, 1991; Fajardo-Somera et al., 2015). These findings suggest that, at least in *N. crassa*, CSE proteins are unlikely to be directly involved in the transport of CHSs unique to filamentous fungi. However, the possibility that CSE interacts with these specific CHS cannot be entirely excluded, as the significant reduction in GlcNAc levels observed in *Δchs-1*, *Δchs-6*, and *Δchs-7* knockout strains (Fajardo-Somera et al., 2015) is similar to the results seen here for the single and double knockout mutants for CSE proteins. Further experiments are needed to investigate the potential relationship between CSE and other CHSs.

The absence of *cse-8* also appeared to affect sexual reproduction in *N. crassa*, resulting in perithecia with two beaks (**Figure 9E**). We termed this phenotype “*shaka*” due to its resemblance to the hand gesture used by surfers in Hawaii. Around 20% of the perithecia produced in homozygous and heterozygous sexual crosses exhibited the shaka phenotype (**Figure 9F**), although this did not affect the production of viable ascospores. Despite there being no previous research on this shaka phenotype, *chs-3* is essential for the production of mature perithecia (Fajardo-Somera et al., 2015), suggesting that the absence of *cse-8* could affect sexual development in *N. crassa*. The developmental timing of mature perithecia does not differ from that of WT fruiting bodies (Lehr et al., 2014). Although *cse-8* expression varies during perithecium development (Lehr et al., 2014), it does not appear to be essential, as functional beaks and viable ascospores are still formed in *Δcse-8* mutants. Therefore, *cse-8* provides an interesting model for studying ostiole biogenesis in ascomycete fungi.

## 4 Conclusions

Our findings demonstrate that CSE proteins play a role in cell wall development and chitin synthesis in *N. crassa*. Also, we could confirm the conserved function of CSE-8 in the ER, and its role in the *de novo* synthesis, and in the transport of CHS-3 to the SPK. This research highlights the need of further investigation to elucidate the transport mechanisms of other CHS, particularly those exclusive to filamentous fungi (CHS classes 3, 5, 6, and 7). The current evidence suggests that the biogenesis of the CHS exclusive to filamentous fungi may involve pathways independent of CSE-mediated receptors.

## Data Availability

The original contributions presented in the study are included in the article/**Supplementary Material**. Further inquiries can be directed to the corresponding author.

## References

[B1] Bartnicki-Garcia S. (1987). Chitosomes and chitin biogenesis. Food Hydrocolloids 1, 353–358. doi: 10.1016/S0268-005X(87)80025-5

[B2] Bartnicki-Garcia S. (2006). Chitosomes: past, present and future. FEMS Yeast Res. 6, 957–965. doi: 10.1111/j.1567-1364.2006.00158.x 16981903

[B3] Bartnicki-Garcia S. Bracker C. E. Reyes E. Ruiz-Herrera J. (1978). Isolation of chitosomes from taxonomically diverse fungi and synthesis of chitin microfibrils *in vitro* . Exp. Mycology 2, 173–192. doi: 10.1016/S0147-5975(78)80031-0

[B4] Bartnicki-Garcia S. Persson J. Chanzy H. (1994). An electron microscope and electron diffraction study of the effect of calcofluor and congo red on the biosynthesis of chitin *in vitro* . Arch. Biochem. Biophysics 310, 6–15. doi: 10.1006/abbi.1994.1133 8161221

[B5] Bolte S. Cordelières F. P. (2006). A guided tour into subcellular colocalization analysis in light microscopy. J. Microscopy 224, 213–232. doi: 10.1111/j.1365-2818.2006.01706.x 17210054

[B6] Borkovich K. A. Alex L. A. Yarden O. Freitag M. Turner G. E. Read N. D.. (2004). Lessons from the genome sequence of *neurospora crassa* : tracing the path from genomic blueprint to multicellular organism. Microbiol. Mol. Biol. Rev. 68, 1–108. doi: 10.1128/MMBR.68.1.1-108.2004 15007097 PMC362109

[B7] Bowman B. J. Abreu S. Margolles-Clark E. Draskovic M. Bowman E. J. (2011). Role of four calcium transport proteins, encoded by *nca-1* , *nca-2* , *nca-3* , and *cax* , in maintaining intracellular calcium levels in *neurospora crassa* . Eukaryotic Cell 10, 654–661. doi: 10.1128/EC.00239-10 21335528 PMC3127652

[B8] Bowman B. J. Draskovic M. Freitag M. Bowman E. J. (2009). Structure and distribution of organelles and cellular location of calcium transporters in *Neurospora crassa* . Eukaryotic Cell 8, 1845–1855. doi: 10.1128/EC.00174-09 19801418 PMC2794220

[B9] Bowman S. M. Free S. J. (2006). The structure and synthesis of the fungal cell wall. BioEssays 28, 799–808. doi: 10.1002/bies.20441 16927300

[B10] Bulik D. A. Olczak M. Lucero H. A. Osmond B. C. Robbins P. W. Specht C. A. (2003). Chitin synthesis in *saccharomyces cerevisiae* in response to supplementation of growth medium with glucosamine and cell wall stress. Eukaryotic Cell 2, 886–900. doi: 10.1128/EC.2.5.886-900.2003 14555471 PMC219353

[B11] Cabib E. (2004). The septation apparatus, a chitin-requiring machine in budding yeast. Arch. Biochem. Biophysics 426, 201–207. doi: 10.1016/j.abb.2004.02.030 15158670

[B12] Cabib E. Schmidt M. (2003). Chitin synthase III activity, but not the chitin ring, is required for remedial septa formation in budding yeast. FEMS Microbiol. Lett. 224, 299–305. doi: 10.1016/S0378-1097(03)00477-4 12892896

[B13] Cabrera-Ponce J. L. León-Ramírez C. G. Verver-Vargas A. Palma-Tirado L. Ruiz-Herrera J. (2012). Metamorphosis of the basidiomycota ustilago maydis: transformation of yeast-like cells into basidiocarps. Fungal Genet. Biol. 49, 765–715. doi: 10.1016/j.fgb.2012.07.005 22921263

[B14] Chen D.-D. Wang Z.-B. Wang L.-X. Zhao P. Yun C.-H. Bai L. (2023). Structure, catalysis, chitin transport, and selective inhibition of chitin synthase. Nat. Commun. 14, 4776. doi: 10.1038/s41467-023-40479-4 37553334 PMC10409773

[B15] Chen W. Cao P. Liu Y. Yu A. Wang D. Chen L.. (2022). Structural basis for directional chitin biosynthesis. Nature 610, 402–408. doi: 10.1038/s41586-022-05244-5 36131020 PMC9556331

[B16] Davis R. H. (2000). Neurospora: contributions of a model organism. Oxford New York: Oxford University Press.

[B17] Dharwada S. T. Dalton L. E. Bean B. D. M. Padmanabhan N. Choi C. Schluter C.. (2018). The chaperone chs7 forms a stable complex with chs3 and promotes its activity at the cell surface. Traffic 19, 285–295. doi: 10.1111/tra.12553 29405545

[B18] Fajardo-Somera R. A. Jöhnk B. Bayram Ö Valerius O. Braus G. H. Riquelme M. (2015). Dissecting the function of the different chitin synthases in vegetative growth and sexual development in *neurospora crassa* . Fungal Genet. Biol. 75, 30–45. doi: 10.1016/j.fgb.2015.01.002 25596036

[B19] Fischer-Parton S. Parton R. M. Hickey P. C. Dijksterhuis J. Atkinson H. A. Read N. D. (2000). Confocal microscopy of FM4-64 as a tool for analysing endocytosis and vesicle trafficking in living fungal hyphae. J. Microscopy 198, 246–595. doi: 10.1046/j.1365-2818.2000.00708.x 10849201

[B20] González Montoro A. Chumpen Ramirez S. Quiroga R. Valdez Taubas J. (2011). Specificity of transmembrane protein palmitoylation in yeast. PloS One 6, e16969. doi: 10.1371/journal.pone.0016969 21383992 PMC3044718

[B21] Gow N. A. R. Latge J.-P. Munro C. A. (2017). The fungal cell wall: structure, biosynthesis, and function. Edited by joseph heitman. Microbiol. Spectr. 5, 5.3.01. doi: 10.1128/microbiolspec.FUNK-0035-2016 PMC11687499 28513415

[B22] Hendershot L. M. Wei J. Y. Gaut J. R. Lawson B. Freiden P. J. Murti K. G. (1995). *In vivo* expression of mammalian biP ATPase mutants causes disruption of the endoplasmic reticulum. Mol. Biol. Cell 6, 283–296. doi: 10.1091/mbc.6.3.283 7612964 PMC301188

[B23] Hernández-González M. Bravo-Plaza I. Pinar M. De Los Ríos V. Arst H. N. Peñalva M. A. (2018). Endocytic recycling via the TGN underlies the polarized hyphal mode of life. PloS Genet. 14, e1007291. doi: 10.1371/journal.pgen.1007291 29608571 PMC5880334

[B24] Hickey P. C. Jacobson D. J. Read N. D. Glass N. L. (2002). Live-cell imaging of vegetative hyphal fusion in *neurospora crassa* . Fungal Genet. Biol. 37, 109–119. doi: 10.1016/S1087-1845(02)00035-X 12223195

[B25] Hickey P. C. Swift S. R. Roca M. G. Read N. D. (2004). Live-cell imaging of filamentous fungi using vital fluorescent dyes and confocal microscopy. In. Methods Microbiol. 34, 63–87. doi: 10.1016/S0580-9517(04)34003-1

[B26] Honda S. Selker E. U. (2009). Tools for fungal proteomics: multifunctional neurospora vectors for gene replacement, protein expression and protein purification. Genetics 182, 11–235. doi: 10.1534/genetics.108.098707 19171944 PMC2674810

[B27] Jin J. Iwama R. Takagi K. Horiuchi H. (2021). AP-2 complex contributes to hyphal-tip-localization of a chitin synthase in the filamentous fungus aspergillus nidulans. Fungal Biol. 125, 806–145. doi: 10.1016/j.funbio.2021.05.009 34537176

[B28] Jumper J. Evans R. Pritzel A. Green T. Figurnov M. Ronneberger O.. (2021). Highly accurate protein structure prediction with alphaFold. Nature 596, 583–589. doi: 10.1038/s41586-021-03819-2 34265844 PMC8371605

[B29] Kappel L. Münsterkötter M. Sipos G. Escobar Rodriguez C. Gruber S. (2020). Chitin and chitosan remodeling defines vegetative development and *trichoderma* biocontrol. Edited by alex andrianopoulos. PloS Pathog. 16, e1008320. doi: 10.1371/journal.ppat.1008320 32078661 PMC7053769

[B30] Kar B. Patel P. Ao J. Free S. J. (2019). Neurospora crassa family GH72 glucanosyltransferases function to crosslink cell wall glycoprotein N-linked galactomannan to cell wall lichenin. Fungal Genet. Biol. 123, 60–69. doi: 10.1016/j.fgb.2018.11.007 30503329

[B31] Karamanos T. K. Tugarinov V. Clore G.M. (2020). An S/T motif controls reversible oligomerization of the hsp40 chaperone DNAJB6b through subtle reorganization of a β Sheet backbone. Proc. Natl. Acad. Sci. 117, 30441–30505. doi: 10.1073/pnas.2020306117 33199640 PMC7720152

[B32] Knafler H. C. Smaczynska-de Rooij I. I. Walker L. A. Lee K. K. Gow N. A. R. Ayscough K. R. (2019). AP-2-dependent endocytic recycling of the chitin synthase chs3 regulates polarized growth in *candida albicans* . mBio 10, e02421–e02418. doi: 10.1128/mBio.02421-18 30890602 PMC6426607

[B33] Kota J. Ljungdahl P. O. (2005). Specialized membrane-localized chaperones prevent aggregation of polytopic proteins in the ER. J. Cell Biol. 168, 79–88. doi: 10.1083/jcb.200408106 15623581 PMC2171667

[B34] Kota J. Gilstring C. F. Ljungdahl P. O. (2007). Membrane chaperone Shr3 assists in folding amino acid permeases preventing precocious ERAD. The Journal of Cell Biology 176, 617–628. doi: 10.1083/jcb.200612100 17325204 PMC2064020

[B35] Lajoie P. Moir R. D. Willis I. M. Snapp E. L. (2012). Kar2p availability defines distinct forms of endoplasmic reticulum stress in living cells. Mol. Biol. Cell 23, 955–964. doi: 10.1091/mbc.e11-12-0995 22219379 PMC3290652

[B36] Lam K. K. Y. Davey M. Sun B. Roth A. F. Davis N. G. Conibear E. (2006). Palmitoylation by the DHHC protein pfa4 regulates the ER exit of chs3. J. Cell Biol. 174, 19–25. doi: 10.1083/jcb.200602049 16818716 PMC2064155

[B37] Lau W.-T. W. Howson R. W. Malkus P. Schekman R. O’Shea E. K. (2000). Pho86p, an endoplasmic reticulum (ER) resident protein in *saccharomyces cerevisiae* , is required for ER exit of the high-affinity phosphate transporter pho84p. Proc. Natl. Acad. Sci. 97, 1107–1112. doi: 10.1073/pnas.97.3.1107 10655492 PMC15537

[B38] Lehr N. A. Wang Z. Li N. Hewitt D. A. López-Giráldez F. Trail F.. (2014). Gene expression differences among three *neurospora* species reveal genes required for sexual reproduction in *neurospora crassa* . PloS One 9, e110398. doi: 10.1371/journal.pone.0110398 25329823 PMC4203796

[B39] Letunic I. Bork P. (2024). Interactive tree of life (iTOL) V6: recent updates to the phylogenetic tree display and annotation tool. Nucleic Acids Res. 52(W1):W78–W82. doi: 10.1093/nar/gkae268 PMC11223838 38613393

[B40] Martínez-Andrade J. M. Roberson R. W. Riquelme M. (2024). A bird’s-eye view of the endoplasmic reticulum in filamentous fungi. Microbiol. Mol. Biol. Rev. 88, e00027–e00023. doi: 10.1128/mmbr.00027-23 38372526 PMC10966943

[B41] Monnerjahn C. Techel D. Meyer U. Rensing L. (2001). The grp78 promoter of neurospora crassa: constitutive, stress and differentiation-dependent protein-binding patterns. Curr. Genet. 39, 319–326. doi: 10.1007/s002940100202 11525405

[B42] Morgan W. T. J. Elson L. A. (1934). A colorimetric method for the determination of N-acetylglucosamine and N-acetylchrondrosamine. Biochem. J. 28, 988–955. doi: 10.1042/bj0280988 16745491 PMC1253291

[B43] Munro C. A. (2013). Chitin and glucan, the yin and yang of the fungal cell wall, implications for antifungal drug discovery and therapy. In. Adv. Appl. Microbiol. 83, 145–172. doi: 10.1016/B978-0-12-407678-5.00004-0 23651596

[B44] Munro C. A. Winter K. Buchan A. Henry K. Becker J. M. Brown A. J. P.. (2001). Chs1 of *candida albicans* is an essential chitin synthase required for synthesis of the septum and for cell integrity. Mol. Microbiol. 39, 1414–1426. doi: 10.1046/j.1365-2958.2001.02347.x 11251855

[B45] Orci L. Perrelet A. Ravazzola M. Wieland F. T. Schekman R. Rothman J. E. (1993). BFA bodies: A subcompartment of the endoplasmic reticulum. Proc. Natl. Acad. Sci. United States America 90, 11089–11093. doi: 10.1073/pnas.90.23.11089 PMC47927 8248213

[B46] Orlean P. (2012). Architecture and biosynthesis of the *saccharomyces cerevisiae* cell wall. Genetics 192, 775–818. doi: 10.1534/genetics.112.144485 23135325 PMC3522159

[B47] Pacheco-Arjona J. R. Ramirez-Prado J. H. (2014). Large-scale phylogenetic classification of fungal chitin synthases and identification of a putative cell-wall metabolism gene cluster in *aspergillus* genomes. PloS One 9, e104920. doi: 10.1371/journal.pone.0104920 25148134 PMC4141765

[B48] Pammer M. Briza P. Ellinger A. Schuster T. Stucka R. Feldmann H.. (1992). *DIT101 (CSD2, CAL1)* , a cell cycle-regulated yeast gene required for synthesis of chitin in cell walls and chitosan in spore walls. Yeast 8, 1089–1099. doi: 10.1002/yea.320081211 1293886

[B49] Preechasuth K. Anderson J. C. Peck S. C. Brown A. J. P. Gow N. A. R. Lenardon M. D. (2015). Cell wall protection by the *candida albicans* class I chitin synthases. Fungal Genet. Biol. 82, 264–276. doi: 10.1016/j.fgb.2015.08.001 26257018 PMC4557417

[B50] Ram A. F.J. Klis F. M. (2006). Identification of fungal cell wall mutants using susceptibility assays based on calcofluor white and congo red. Nat. Protoc. 1, 2253–2565. doi: 10.1038/nprot.2006.397 17406464

[B51] Ren Z. Chhetri A. Guan Z. Suo Y. Yokoyama K. Lee S.-Y. (2022). Structural basis for inhibition and regulation of a chitin synthase from candida albicans. Nat. Struct. Mol. Biol. 29, 653–645. doi: 10.1038/s41594-022-00791-x 35788183 PMC9359617

[B52] Rico-Ramírez A. M. Roberson R. W. Riquelme M. (2018). Imaging the secretory compartments involved in the intracellular traffic of CHS-4, a class IV chitin synthase. Neurospora Crassa. Fungal Genet. Biol. 117, 30–42. doi: 10.1016/j.fgb.2018.03.006 29601947

[B53] Riquelme M. (2013). Tip growth in filamentous fungi: A road trip to the apex. Annu. Rev. Microbiol. 67, 587–609. doi: 10.1146/annurev-micro-092412-155652 23808332

[B54] Riquelme M. Bartnicki-García S. (2008). Advances in understanding hyphal morphogenesis: ontogeny, phylogeny and cellular localization of chitin synthases. Fungal Biol. Rev. 22, 56–70. doi: 10.1016/j.fbr.2008.05.003

[B55] Riquelme M. Bartnicki-García S. González-Prieto J. M. Sánchez-León E. Verdín-Ramos J. A. Beltrán-Aguilar A.. (2007). Spitzenkörper localization and intracellular traffic of green fluorescent protein-labeled CHS-3 and CHS-6 chitin synthases in living hyphae of *neurospora crassa* . Eukaryotic Cell 6, 1853–1864. doi: 10.1128/EC.00088-07 17644657 PMC2043383

[B56] Sacristan C. Manzano-Lopez J. Reyes A. Spang A. Muñiz M. Roncero C. (2013). Oligomerization of the chitin synthase chs 3 is monitored at the G olgi and affects its endocytic recycling. Mol. Microbiol. 90, 252–266. doi: 10.1111/mmi.12360 23926947

[B57] Sánchez‐León E. Bowman B. Seidel C. Fischer R. Novick P. Riquelme M. (2014). The Rab GTPase YPT-1 associates with Golgi cisternae and Spitzenkörper microvesicles in *Neurospora crassa* . Mol. Microbiol. 95, 472–490. doi: 10.1111/mmi.12878 25425138

[B58] Sánchez-León E. Verdín J. Freitag M. Roberson R. W. Bartnicki-Garcia S. Riquelme M. (2011). Traffic of chitin synthase 1 (CHS-1) to the spitzenkörper and developing septa in hyphae of *neurospora crassa*: actin dependence and evidence of distinct microvesicle populations. Eukaryotic Cell 10, 683–695. doi: 10.1128/EC.00280-10 21296914 PMC3127655

[B59] Sanz M. (2004). *Saccharomyces cerevisiae* bni4p directs the formation of the chitin ring and also participates in the correct assembly of the septum structure. Microbiology 150, 3229–3241. doi: 10.1099/mic.0.27352-0 15470103

[B60] Sanz M. Carrano L. Jiménez C. Candiani G. Trilla J. A. Durán A.. (2005). *Candida albicans* strains deficient in CHS7, a key regulator of chitin synthase III, exhibit morphogenetic alterations and attenuated virulence. Microbiology 151, 2623–2636. doi: 10.1099/mic.0.28093-0 16079341

[B61] Seaman M. N. J. (2008). Membrane traffic in the secretory pathway: endosome protein sorting: motifs and machinery. Cell. Mol. Life Sci. 65, 2842–2858. doi: 10.1007/s00018-008-8354-1 18726175 PMC11131859

[B62] Shen M. W. Y. Shah D. Chen W. Da Silva N. (2012). Enhanced arsenate uptake in *saccharomyces cerevisiae* overexpressing the pho84 phosphate transporter. Biotechnol. Prog. 28, 654–661. doi: 10.1002/btpr.1531 22628173

[B63] Sherwood P. W. Carlson M. (1999). Efficient export of the glucose transporter hxt1p from the endoplasmic reticulum requires gsf2p. Proc. Natl. Acad. Sci. 96, 7415–7420. doi: 10.1073/pnas.96.13.7415 10377429 PMC22100

[B64] Smith K. M. Phatale P. A. Sullivan C. M. Pomraning K. R. Freitag M. (2011). Heterochromatin is required for normal distribution of *neurospora crassa* cenH3. Mol. Cell. Biol. 31, 2528–2542. doi: 10.1128/MCB.01285-10 21505064 PMC3133421

[B65] Starr T. L. Pagant S. Wang C. W. Schekman R. (2012). Sorting signals that mediate traffic of chitin synthase III between the TGN/endosomes and to the plasma membrane in yeast. PloS One 7, e46386. doi: 10.1371/journal.pone.0046386 23056294 PMC3463608

[B66] Sudoh M. Tatsuno K. Ono N. Ohta A. Chibana H. Yamada-Okabe H.. (1999). The *candida albicans* CHS4 gene complements a *saccharomyces cerevisiae* skt5/chs4 mutation and is involved in chitin biosynthesis. Microbiology 145, 1613–1622. doi: 10.1099/13500872-145-7-1613 10439400

[B67] Sun Y. MacRae T. H. (2005). Small heat shock proteins: molecular structure and chaperone function. Cell. Mol. Life Sci. 62, 2460–2476. doi: 10.1007/s00018-005-5190-4 16143830 PMC11138385

[B68] Torres-García E. Pinto-Cámara R. Linares A. Martínez D. Abonza V. Brito-Alarcón E.. (2022). Extending resolution within a single imaging frame. Nat. Commun. 13, 7452. doi: 10.1038/s41467-022-34693-9 36460648 PMC9718789

[B69] Travers K. J. Patil C. K. Wodicka L. Lockhart D. J. Weissman J. S. Walter P. (2000). Functional and genomic analyses reveal an essential coordination between the unfolded protein response and ER-associated degradation. Cell 101, 249–258. doi: 10.1016/S0092-8674(00)80835-1 10847680

[B70] Trilla J. A. Durán A. Roncero C. (1999). Chs7p, a new protein involved in the control of protein export from the endoplasmic reticulum that is specifically engaged in the regulation of chitin synthesis in *saccharomyces cerevisiae* . J. Cell Biol. 145, 1153–1163. doi: 10.1083/jcb.145.6.1153 10366589 PMC2133151

[B71] Vogel H. J. (1956). A convenient growth medium for neurospora (Medium N). Microbial Genet. Bull. 13, 42–43.

[B72] Weichert M. Guirao-Abad J. Aimanianda V. Krishnan K. Grisham C. Snyder P.. (2020). Functional coupling between the unfolded protein response and endoplasmic reticulum/golgi ca^2+^-ATPases promotes stress tolerance, cell wall biosynthesis, and virulence of *aspergillus fumigatus.* Edited by J. Andrew alspaugh. mBio 11, e01060–e01020. doi: 10.1128/mBio.01060-20 32487759 PMC7267887

[B73] Yan Y. Tao H. He Ji Huang S.-Y. (2020). The HDOCK server for integrated protein–Protein docking. Nat. Protoc. 15, 1829–1525. doi: 10.1038/s41596-020-0312-x 32269383

[B74] Yarden O. Yanofsky C. (1991). Chitin synthase 1 plays a major role in cell wall biogenesis. Neurospora Crassa. Genes Dev. 5, 2420–2430. doi: 10.1101/gad.5.12b.2420 1836444

