# Supplementary material for: CSE-8, a filamentous fungus-specific Shr3-like chaperone, facilitates endoplasmic reticulum exit of chitin synthase CHS-3 (class I) in Neurospora crassa
Source: Front Fungal Biol. 2025 Jan 24;5:1505388. doi: 10.3389/ffunb.2024.1505388 (PMC11803449; doi:10.3389/ffunb.2024.1505388)
Supplement: Supplementary file 1 [file DataSheet1.docx]

Supplementary Material

# Supplementary Figures

**Supplementary Figure 1.** Molecular characterization of the strain NSSG6. A) Diagram of the molecular cassette *pccg-1::chs-3::gfp*. The diagram shows the primers used to amplify the fragment corresponding to the *pccg-1* promoter and the first 1412 bp of the *chs-3* open reading frame (ORF) (NCU04251; left) and the fragment completing the *chs-3* ORF (1440 bp) plus the *gfp* fluorescent protein gene (right). B) Representative diagram of the *cse-8* ORF (NCU01814) with the primers used to verify the gene mutation in the strain. (C) Electrophoresis gels of the PCR amplification of the F1 and F2 fragments shown in A. (D) Electrophoresis gel of the PCR reaction of *cse8* using genomic DNA from the strain NSSG6 as the template (a), and from the FGSC#4200 strain as a positive control of the amplification (b).

**Supplementary Figure 2.** CSE-8 and CSE-7 are not essential for the subcellular transport of CHS-1-GFP and CHS-5-GFP. Knockout mutations in *cse-7* and *cse-8* genes do not affect the localization of class III and V CHS to the SPK. Micrographs show strains with endogenous labeling of CHS-1-GFP and CHS-5-GFP in a *∆mus-51::bar*^+^ background (Strain #9718). Scale bars = 10 μm.

**Supplementary Figure 3.** *In silico* model of the interaction between CHS-3 and CSE-8 in *N. crassa*. (A) Membrane distribution of CSE-8 and (B) CHS-3-dimer proteins as suggested by the InterPro database. Amino acid residues LPLC, AVGR, FAAGQV, and ICDG are conserved regions shared with CSE-7 and Chs7. (C) A model of the interaction between CSE-8 and CHS-3 dimers with suggested amino acid interactions is shown in deep blue and orange. The transmembrane region of CSE-8 is highlighted in yellow-orange. (D) A detailed view of the interactive region between CSE-8 and CHS-3 shows the suggested amino acid interactions. Arrows point to the TM domains for CSE-8 (left) and CHS-3 (right). (E) Zoomed-in regions of the transmembrane interactions are depicted in (C). Magenta dotted lines indicate distances <4 Å between residues.

**Supplementary Figure 4. Molecular dockings of CHS-4/CSE-8, CHS-3/CSE-7 and CHS-4/CSE-7.** (A) CHS-3 dimer structure and protein region distribution. The structure of the CHS-3 dimer is depicted with the following color coding: disordered regions are colored in green; the catalytic CHS domain is shown in pink; transmembrane regions are in purple, and cytosolic and non-cytoplasmic regions are in yellow. One of the dimer's subunits is colored in gray. (B) Molecular docking between CSE-8 (green) and CHS-4 (cyan) was used as a negative control of CHS-3/CSE-8 molecular docking. The interactions detected by both proteins are shown in magenta. (C) Molecular docking between CSE-7 (cyan) and CHS-3 dimer (green). Only two interaction sites (colored in magenta) were detected. (D) Molecular docking of CHS-4 (green) and CSE-7 (cyan/salmon), which was considered a positive control. Interaction sites are marked in magenta and pointed with white arrows. Close-up of the region marked with a yellow box (E) and in navy blue (F) in C. All interactions are marked with yellow dashes. Note that the regions and number of interactions are larger and more numerous than in the negative controls.

**Supplementary Figure 5**. Effect of ER stressors on the subcellular localization of RFP-BiP, CSE-7-GFP, and CHS-4-GFP. A) Heterokaryon strain (NSSG3) expressing CSE-8-GFP and RFP-BiP was exposed to DTT (1.25 mM). CSE-8 accumulates in clusters along with BiP (merged channel) independently and is also observed at the apical PM. (B) Co-localization plots and Manders' coefficients of CSE-8-GFP and RFP-BiP shown in panel A. (C) Confocal laser scanning microscopy of the NSSG3 strain stained with FM4-64, grown in media with DTT. CSE-8-GFP co-localizes with FM4-64-stained endomembranes (white regions and blue arrows). The distribution of the outer layer (stained with FM4-64) and the inner layer containing CSE-8-GFP indicates complete disruption of the SPK at the hyphal tip. (D) Co-localization plots and Pearson’s coefficient of CSE-8-GFP and FM-464 of panel C micrographs. (E-F) Changes in the intracellular distribution of CSE-7-GFP and CHS-4-GFP in hyphae exposed to DTT. Both proteins accumulate in subapical areas, and tubular structures disappear in DTT-exposed hyphae. (F) Distribution of RFP-BiP in hyphae with and without TM (4.25 μg/mL) treatment. RFP-BiP accumulates in region II of the hyphae post-treatment. (G) Fluorescence intensity plots of the hyphae shown in panel F. RFP-BiP fluorescence intensity is higher in the first 22 μm of the TM-treated hyphae compared to untreated hyphae, where fluorescence is more distributed beyond the first 20 μm. Yellow arrows indicate the presence of GFP-tagged proteins at the hyphal tip. Scale bars = 10 μm.

**Supplementary Figure 6**. Effect of DTT stressors on the subcellular localization of CSE-7-GFP (A), and CHS-4-GFP (B). Scale bars = 10 μm.

# Supplementary videos

Supplementary Video 1. Subcellular distribution of CSE-8-GFP by LSCM. Scale bar = 10 $\mu$m

Supplementary Video 2. Dynamics of CSE-8-GFP and CSE-7-mCherry by SDCM. Scale bar = 10 $\mu$m
