# Supplementary figures and images for: CSE-8, a filamentous fungus-specific Shr3-like chaperone, facilitates endoplasmic reticulum exit of chitin synthase CHS-3 (class I) in Neurospora crassa
Source: Front Fungal Biol. 2025 Jan 24;5:1505388. doi: 10.3389/ffunb.2024.1505388 (PMC11803449; doi:10.3389/ffunb.2024.1505388)

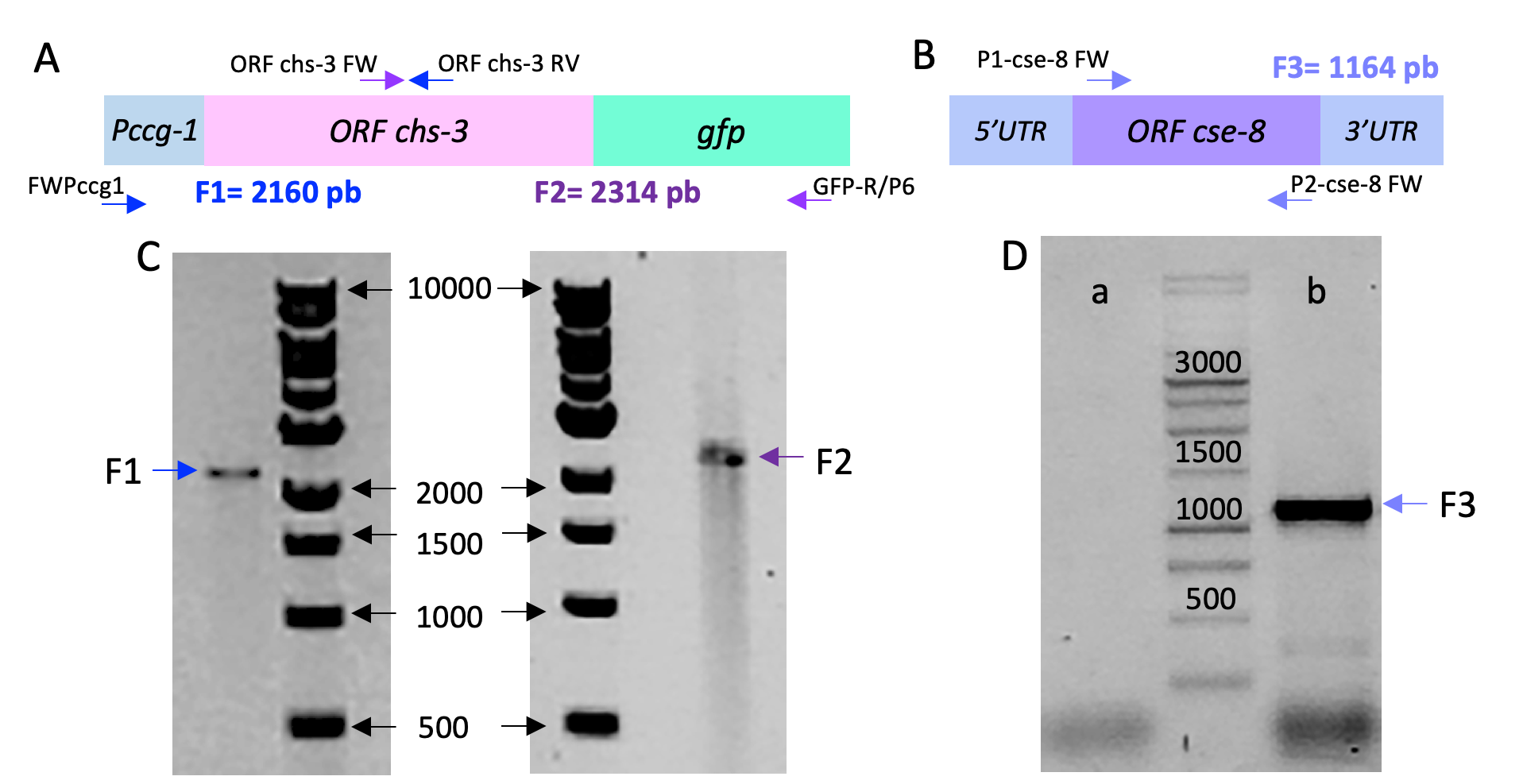

Supplement: Supplementary file 2 [file Image1.tif]

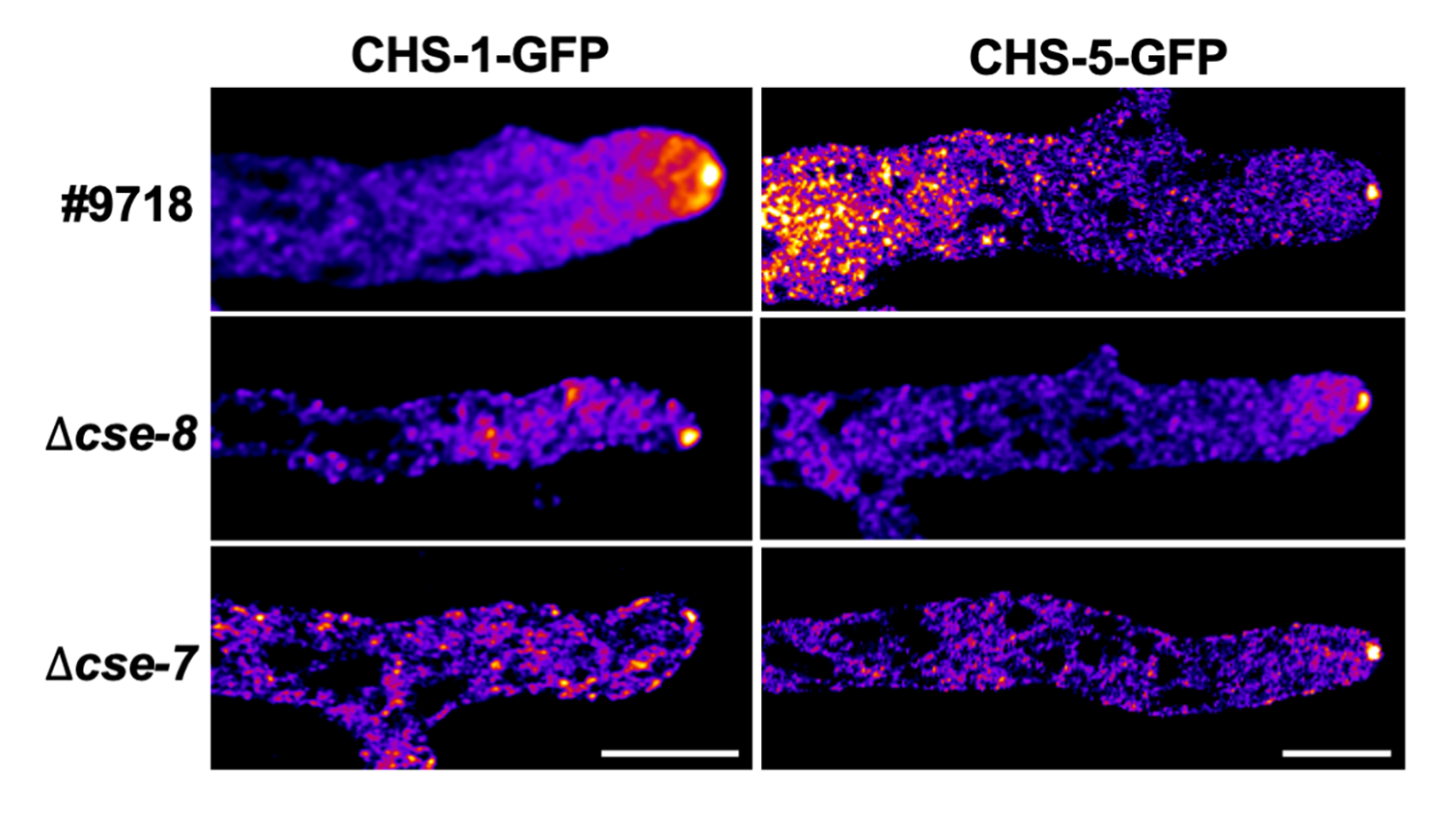

Supplement: Supplementary file 3 [file Image2.tif]

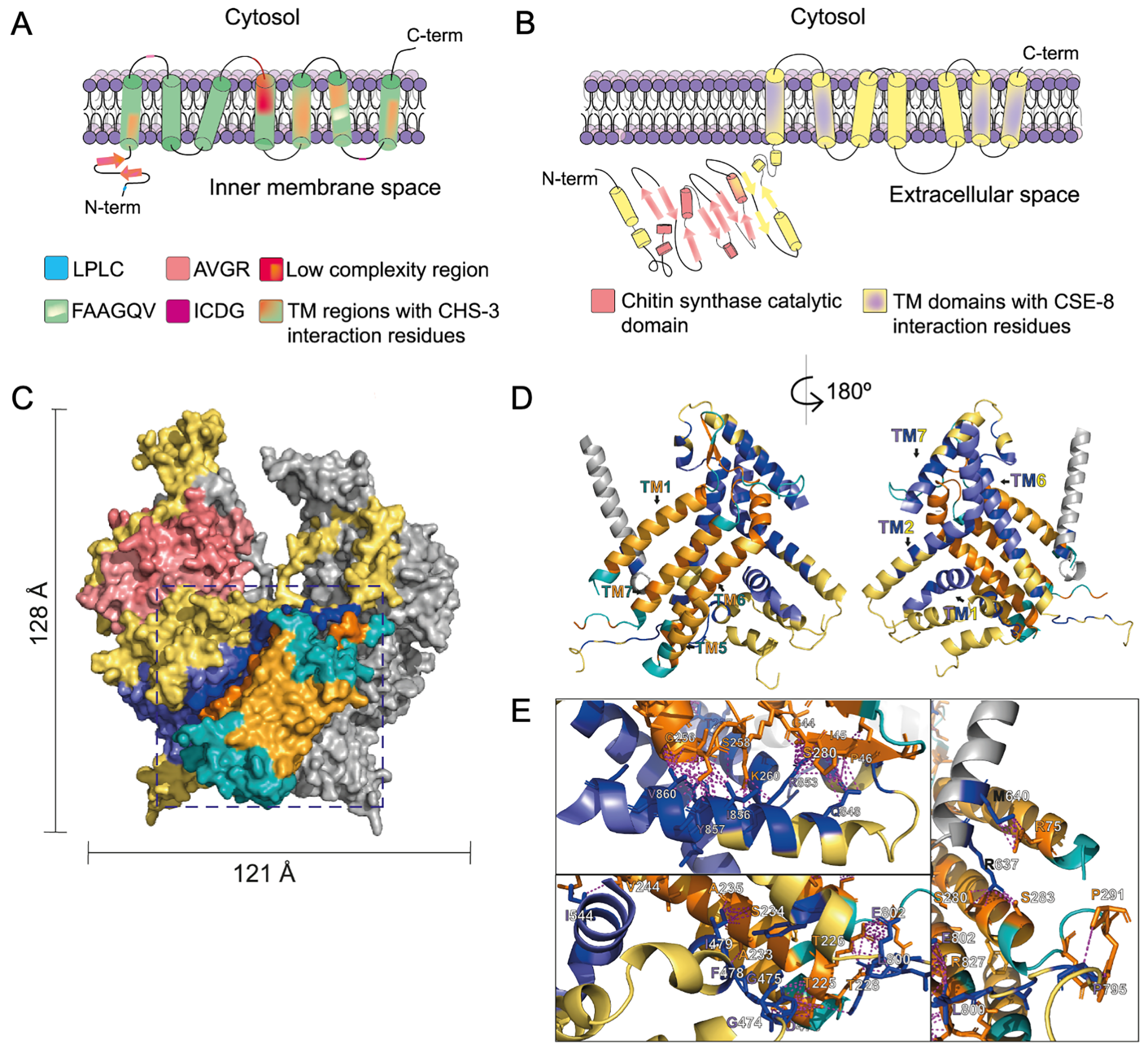

Supplement: Supplementary file 4 [file Image3.tiff]

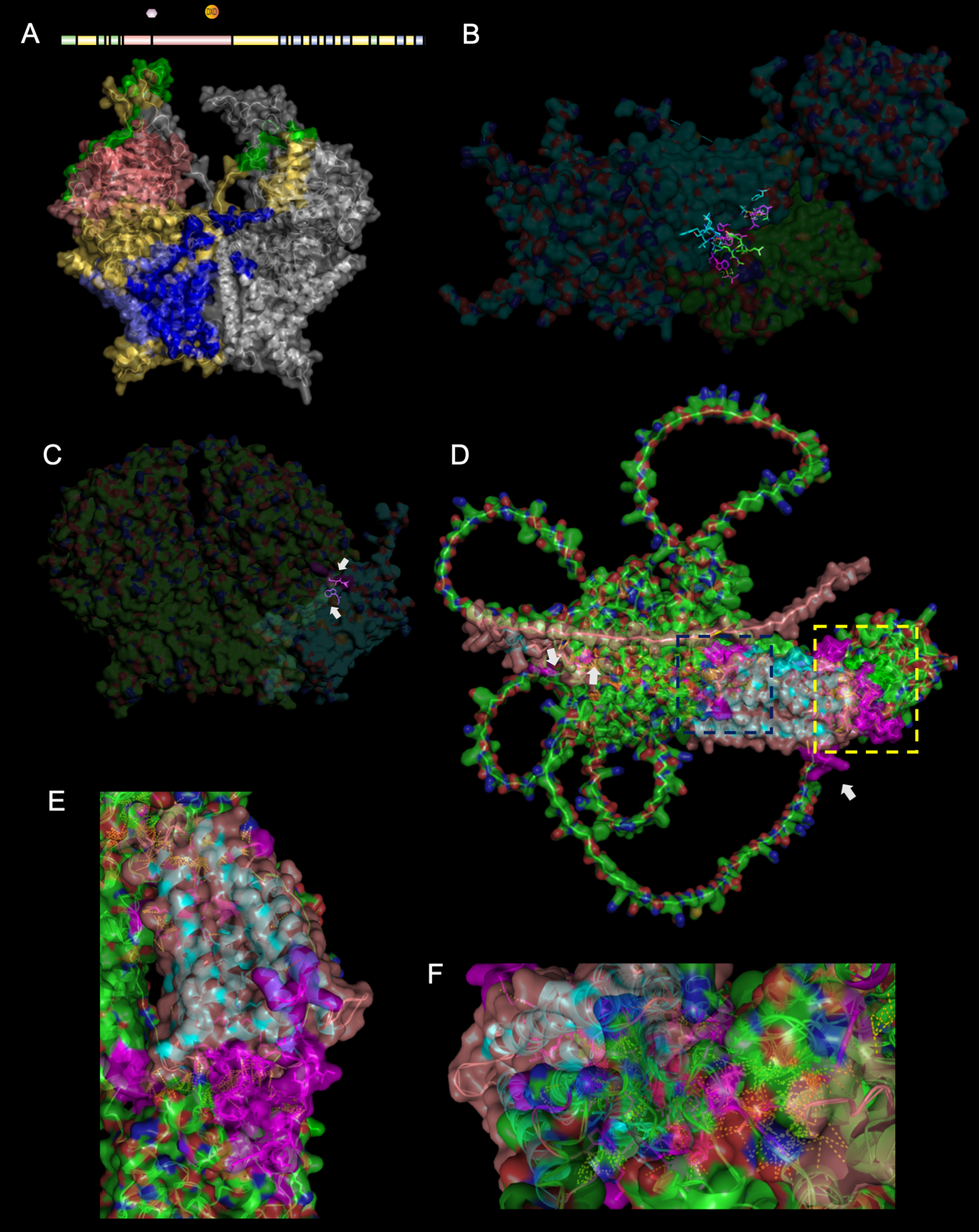

Supplement: Supplementary file 5 [file Image4.tiff]

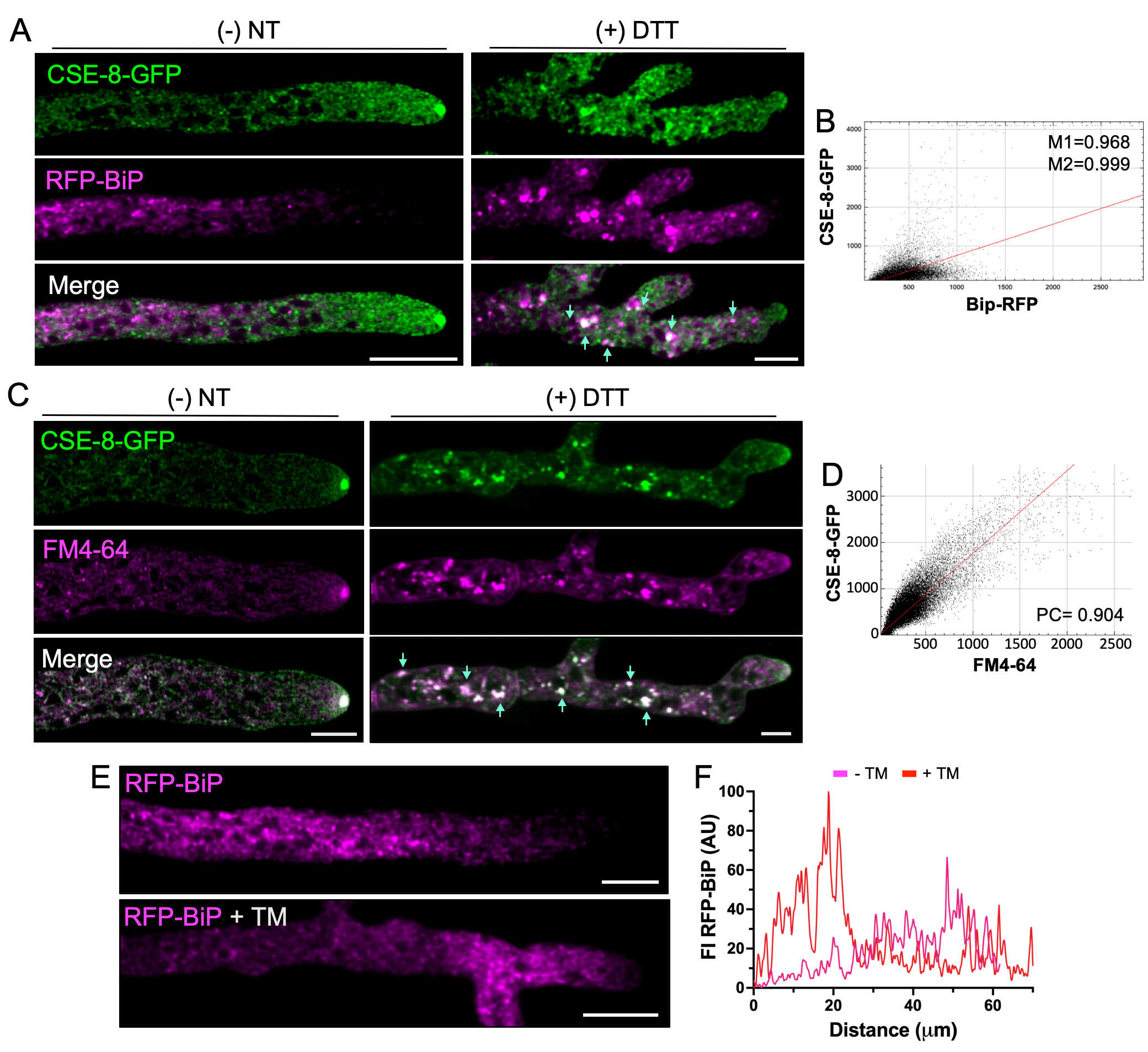

Supplement: Supplementary file 6 [file Image5.tif]

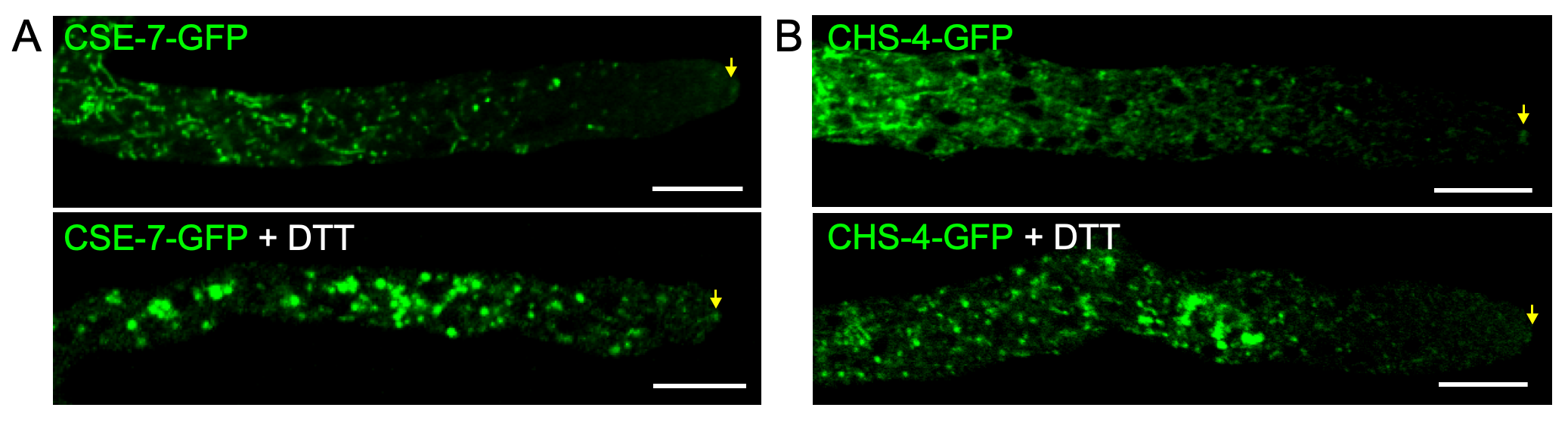

Supplement: Supplementary file 7 [file Image6.tif]
